# MIF-Mediated Hemodilution Promotes Pathogenic Anemia in Experimental African Trypanosomosis

**DOI:** 10.1371/journal.ppat.1005862

**Published:** 2016-09-15

**Authors:** Benoît Stijlemans, Lea Brys, Hannelie Korf, Pawel Bieniasz-Krzywiec, Amanda Sparkes, Liese Vansintjan, Lin Leng, Nele Vanbekbergen, Massimiliano Mazzone, Guy Caljon, Jan Van Den Abbeele, Steven Odongo, Carl De Trez, Stefan Magez, Jo A. Van Ginderachter, Alain Beschin, Richard Bucala, Patrick De Baetselier

**Affiliations:** 1 Laboratory of Cellular and Molecular Immunology, Vrije Universiteit Brussel (VUB), Brussels, Belgium; 2 Lab of Myeloid Cell Immunology, VIB Inflammation Research Center, Ghent, Belgium; 3 Translational Research Center for Gastrointestinal Disorders (TARGID), Department of Clinical and Experimental Medicine, KU Leuven, Leuven, Belgium; 4 VIB Vesalius Research Center, University of Leuven (KUL), Leuven, Belgium; 5 Department of Internal Medicine, Yale University School of Medicine, New Haven, Connecticut, United States of America; 6 Laboratorium Cellulaire Genetica (CEGE), Vrije Universiteit Brussel (VUB), Brussels, Belgium; 7 Laboratory for Microbiology, Parasitology and Hygiene (LMPH), University of Antwerp, Wilrijk, Belgium; 8 Unit of Veterinary Protozoology, Department of Biomedical Sciences, Institute of Tropical Medicine Antwerp (ITM), Antwerp, Belgium; 9 Department of Structural Biology, Vrije Universiteit Brussel, Brussels, Belgium; 10 Laboratory for Biomedical Research, Ghent University Global Campus, Yeonsu-Gu, Incheon, South Korea; University of California, Los Angeles, UNITED STATES

## Abstract

Animal African trypanosomosis is a major threat to the economic development and human health in sub-Saharan Africa. *Trypanosoma congolense* infections represent the major constraint in livestock production, with anemia as the major pathogenic lethal feature. The mechanisms underlying anemia development are ill defined, which hampers the development of an effective therapy. Here, the contribution of the erythropoietic and erythrophagocytic potential as well as of hemodilution to the development of *T*. *congolense*-induced anemia were addressed in a mouse model of low virulence relevant for bovine trypanosomosis. We show that in infected mice, splenic extramedullary erythropoiesis could compensate for the chronic low-grade type I inflammation-induced phagocytosis of senescent red blood cells (RBCs) in spleen and liver myeloid cells, as well as for the impaired maturation of RBCs occurring in the bone marrow and spleen. Rather, anemia resulted from hemodilution. Our data also suggest that the heme catabolism subsequent to sustained erythrophagocytosis resulted in iron accumulation in tissue and hyperbilirubinemia. Moreover, hypoalbuminemia, potentially resulting from hemodilution and liver injury in infected mice, impaired the elimination of toxic circulating molecules like bilirubin. Hemodilutional thrombocytopenia also coincided with impaired coagulation. Combined, these effects could elicit multiple organ failure and uncontrolled bleeding thus reduce the survival of infected mice. MIF (macrophage migrating inhibitory factor), a potential pathogenic molecule in African trypanosomosis, was found herein to promote erythrophagocytosis, to block extramedullary erythropoiesis and RBC maturation, and to trigger hemodilution. Hence, these data prompt considering MIF as a potential target for treatment of natural bovine trypanosomosis.

## Author Summary

Bovine African trypanosomosis is a parasitic disease of veterinary importance that adversely affects the public health and economic development of sub-Saharan Africa. Anemia is a major cause of death associated with this disease. Yet, the mechanisms underlying anemia development are not elucidated, which hampers the design of effective therapeutic strategies. We show here that in a *Trypanosoma congolense* infection mouse model relevant for bovine trypanosomosis, red blood cells (RBCs) are generated in the spleen. This compensates for the impaired maturation of RBCs occurring in the bone marrow, the normal site of RBC generation, and for the destruction of RBCs taking place in the liver and the spleen. Instead, anemia results from an increase in blood volume (hemodilution). The immune molecule Macrophage Migration Inhibitory Factor (MIF) was found to drive RBC destruction, to block RBC maturation, as well as to trigger hemodilution. Iron accumulation in tissue due to sustained RBC destruction and hemodilution causes tissue damage, which culminates in the release of toxic molecules like bilirubin, in impaired production of blood detoxifying molecules like albumin, and in defective coagulation. Combined, these effects initiate multiple organ failure that can reduce the survival of infected mice. Given the unmet medical need for this parasite infection, our findings offer promise for improved treatment protocols in the field.

## Introduction

African trypanosomosis (AT) is a neglected tropical disease of medical and veterinary importance that adversely affects human health and welfare, as well as the economic development in sub-Saharan Africa [1,2]. AT is caused by blood-borne hemoflagellated protozoan parasites from the *Trypanosoma* genus that are transmitted by the hematophagous tsetse fly (*Glossina spp*.) vector [3]. *Trypanosoma-*induced diseases in mammals include sleeping sickness in humans or nagana in domestic livestock, with fatal consequences unless treated [4,5]. Given that so far no effective vaccine is available, that certain trypanosome strains have become resistant to curative and preventive treatments, and that eradication of tsetse flies remains impossible in most regions [1,6], strategies focusing at reducing AT-associated pathogenicity might be an alternative approach to reduce the economic losses in cattle production.

In case of bovine trypanosomosis, the main difference between so-called trypano-susceptible and -tolerant animals relies in their capacity to control anemia development, the major cause of death associated with the disease [7,8], and hereby to remain productive [9]. Differences in erythropoietic potential have been suggested as a contributing factor to anemia development [8]. Yet, the mechanisms underlying this phenomenon remain poorly understood, which hampers the design of effective therapeutic strategies. Given the similarities between the anemic responses of cattle and C57Bl/6 mice upon trypanosome infection [8], the underlying mechanisms were mainly scrutinized in murine models [10,11]. The data collectively suggest that the pro-inflammatory type I immune response, involving TNF, IFN-γ and M1-type (classically activated) myeloid cells, contributes to pathogenicity in general and anemia in particular, and in combination with impaired B-cell functionality, results in reduced survival of the mice [10,12,13]. Thus, identification of gene-products regulating pro-inflammatory signals during the course of the disease might pave the way to develop novel intervention strategies. In this context, we previously identified macrophage migration inhibitory factor (MIF) as a potential susceptibility marker for African trypanosomosis. This ubiquitously produced cytokine is a prominent inducer of systemic inflammation in many inflammatory diseases [14,15] that acts by recruiting and activating myeloid cells towards M1-type cells to the site of inflammation [16,17], and by suppressing apoptosis of inflammatory cells [18]. We have shown using a trypanosusceptible model based on C57Bl/6 mice infected with *T*. *brucei brucei*, that MIF contributes to tissue pathogenicity by sustaining throughout infection a persistent type I pro-inflammatory chemokine (CXCL1, CCL2) and cytokine (IFN-γ, TNF, IL-6) response, and by enhancing the recruitment of Ly6C^+^ monocytes and neutrophils (PMNs) in the liver with concomitant hepatomegaly [19]. Moreover, PMN- but not monocyte-derived MIF was mainly responsible for liver damage. In addition, MIF promotes the development of so-called anemia of inflammation in trypanosusceptible mice by enhancing red blood cell (RBC) clearance from the blood, and by triggering the storage of iron in liver myeloid cells that deprives iron from erythropoiesis and impairs the generation of mature RBCs. However, despite reduced liver injury and anemia levels in *T*. *b*. *brucei*-infected *Mif* ^*-/-*^ mice or mice treated with a neutralizing anti-MIF antibody, the host survival time was not affected [19].

Compared to *T*. *b*. *brucei*, *T*. *congolense* infection in C57Bl/6 mice is considered a trypanotolerant model more relevant for bovine trypanosomosis [20,21]. In contrast to *T*. *b*. *brucei*, *T*. *congolense* causes a chronic infection (3–4 months versus 1 month), due to the capacity of *T*. *congolense*-infected mice to restrain the type I immune response and to switch to an IL-10-mediated tissue protective anti-inflammatory response [21,22]. This model could thus allow a thorough analysis of the mechanisms underlying anemia development in an immune environment different from that of anemia of inflammation. Here, we evaluated the role of MIF in *T*. *congolense* infection-associated anemia development, by focusing on the modulation of the erythropoietic and erythrophagocytic potential in tissues including the bone marrow, the spleen and the liver. Additionally, the contribution of hemodilution to anemia was addressed.

## Results

### 1. MIF deficiency correlates with prolonged survival and reduced tissue pathogenicity during *T*. *congolense* infection

**Fig 1:**
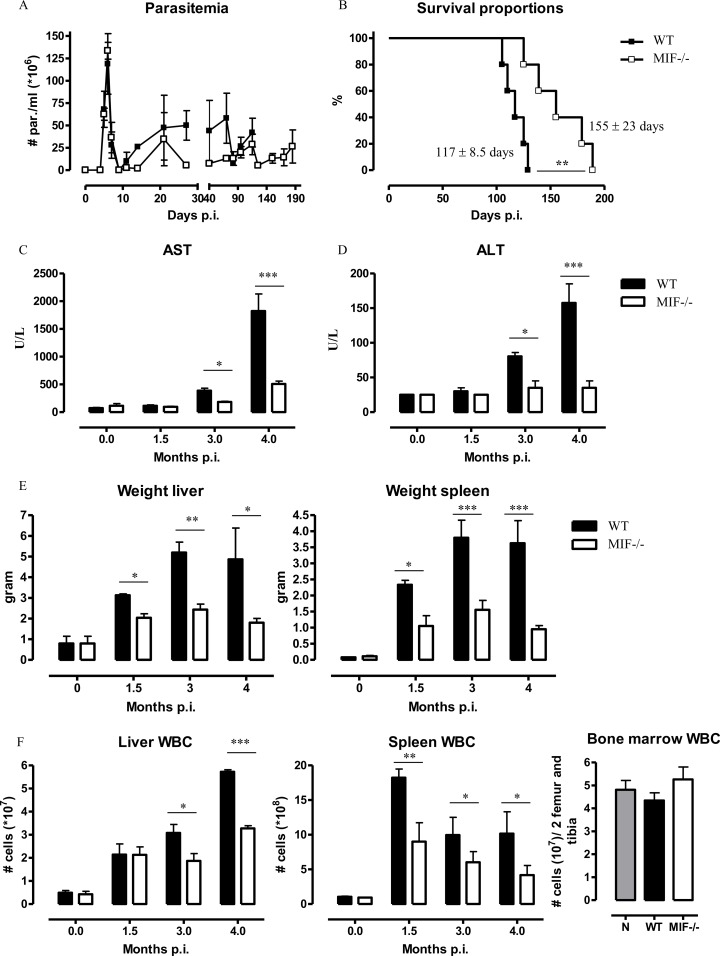
MIF deficiency confers partial protection and reduces hepatosplenomegaly and white blood cell accumulation during *T*. *congolense* infection. **(A)** Parasitemia, **(B)** survival, serum **(C)** AST and **(D)** ALT levels in infected mice. During the course of infection, **(E)** liver and spleen weight as well as **(F)** liver, spleen and bone marrow white blood cell (WBC) numbers. For bone marrow WBCs, only data from 3 months p.i. are shown. Wild type (WT, black symbol); *Mif* ^-/-^ (white symbol) mice. Results are representative of 2 **(E)** or 3 independent experiments and presented as mean **(A, C, D, E, F)** or median **(B)** of 3–5 individual mice ± SEM. *: p≤0.05, **: p≤0.01, ***: p≤0.001.

The systemic production of MIF increased progressively during the course of *T*. *congolense* infection (S1 Fig). Hence, the potential role of MIF in the outcome of i.p. infection was evaluated by comparing wild type (WT) and MIF-deficient (*Mif* ^-/-^) C57Bl/6 mice. Although parasitemia development was similar in WT and *Mif* ^-/-^ mice, a prolongation of median survival time occurred in *Mif* ^-/-^ mice (Fig 1A and 1B). Similar observations were obtained using a natural route, tsetse fly-mediated infection model (S2A and S2B Fig). Considering the similar capacity of WT and *Mif* ^*-/-*^ mice to control parasite growth, the increased survival of *Mif* ^*-/-*^ mice could result from lower tissue pathogenicity [23,24]. In agreement, as compared to WT mice, *Mif* ^*-/-*^ mice exhibited reduced serum AST (alanine aminotransferase, reflecting systemic tissue injury) and ALT (aspartate aminotransferase, reflecting liver injury) levels (Fig 1C and 1D), as well as reduced hepato- and splenomegaly that coincided with a lower increase in the number of white blood cells (WBC) in the liver and the spleen of *Mif* ^*-/-*^ mice (Figs 1E and 1F and S3). No WBC accumulation was observed in the bone marrow of infected WT and *Mif* ^*-/-*^ mice (Fig 1F).

**Fig 2:**
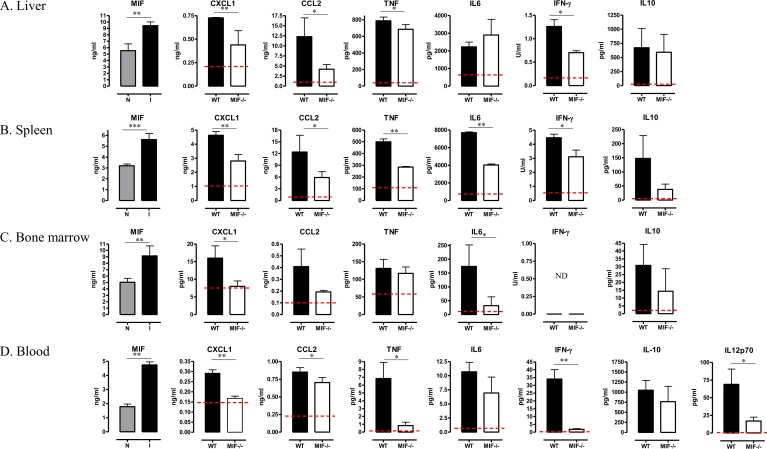
MIF contributes to pathogenic chemokine and cytokine production during *T*. *congolense* infection. At 3 months p.i., *ex-vivo* protein levels of MIF, CXCL1, CCL2, TNF, IL-6, IFN-γ and IL-10 in **(A-C)** supernatant from the liver, spleen, and bone marrow cell cultures, and **(D)** in the blood. IL-12p70 levels in blood are shown for WT (black bar) and *Mif* ^-/-^ (open bar) mice. Protein levels in non-infected mice (dashed line) were similar in both mouse strains. (N.D.: not detectable). Results are representative of 3 independent experiments and presented as mean of 3 individual mice ± SEM. *: p≤0.05, **: p≤0.01, ***: p≤0.001.

The differences in pathogenicity between WT and *Mif* ^*-/-*^ infected mice were clearly established from 3 months post infection (p.i.) (Figs 1C–1F and S3), hence we focused at this time point. At 3 months p.i., MIF secretion was enhanced in the liver, spleen and bone marrow of infected WT mice (Fig 2), mirroring the increased MIF level measured in the blood. In agreement with the reduced tissue injury in infected *Mif* ^*-/-*^ mice, the levels of neutrophil (CXCL1) and monocyte (CCL2) chemoattractants, as well as of pro-inflammatory cytokines documented to contribute to *T*. *congolense*-induced tissue destruction (TNF, IL-6, IFN-γ) [25,26], were increased to a lesser degree in the liver, spleen, bone marrow and blood of *Mif* ^*-/-*^ than WT mice (Fig 2A–2D). This was also true for IL-12p70 in the blood (Fig 2D) [27]. However, the systemic and tissue levels of IL-10, which increases upon *T*. *congolense* infection and is crucial to limit tissue destruction [28], did not differ between WT and *Mif* ^*-/-*^ mice (Fig 2).

**Fig 3:**
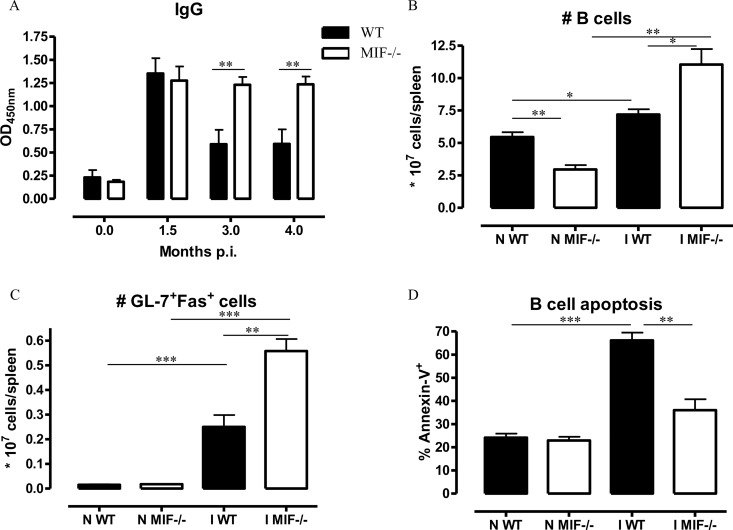
MIF deficiency preserves parasite-specific IgG responses, maintains germinal center formation and prevents B-cell apoptosis during *T*. *congolense* infection. **(A)** Parasite-specific blood IgG level (1/500 dilution) in WT (black bar) and *Mif* ^-/-^ (open bar) mice at 1.5, 3 and 4 months p.i.. At 3 months p.i. **(B)** absolute numbers of B220^+^MHC-II^+^ B-cells and **(C)** of germinal GL-7^+^Fas^+^ B-cells and **(D)** percentage of Annexin-V^+^ B220^+^ cells in the spleen of WT (black bar) and *Mif* ^-/-^ (open bar) mice were determined using the gating strategy described in S5A and S5B Fig. Results are representative of 2 independent experiments and presented as mean of 3 individual mice ± SEM. *: p≤0.05, **: p≤0.01, ***: p≤0.001.

**Fig 4:**
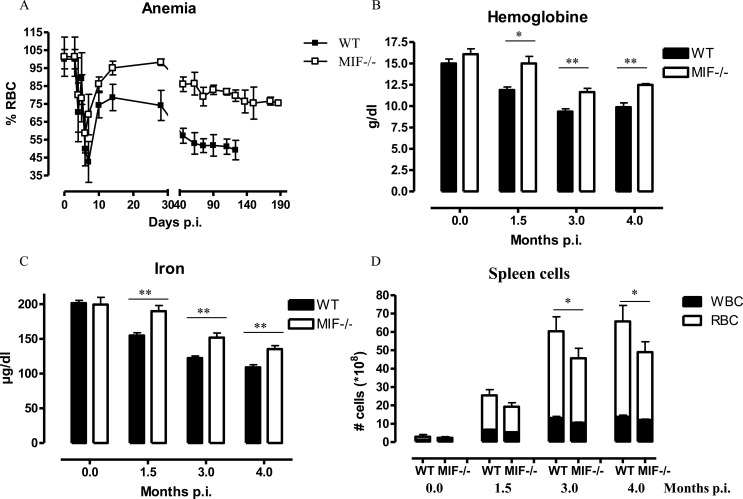
MIF deficiency partially reduces anemia during *T*. *congolense* infection. **(A)** Anemia development in infected WT (closed symbol) and *Mif* ^*-/-*^ (open symbol) mice. Number of RBCs in non-infected mice was set as 100%. At 1.5, 3 and 4 months p.i., **(B)** serum hemoglobin levels, **(C)** serum iron levels, and **(D)** numbers of splenic white blood cells (WBC, black bar) and RBCs (white bar) were determined. Results are representative of 2 independent experiments and presented as mean of 3 individual mice ± SEM. **: p≤0.01, ***: p≤0.001.

In addition to differences in pro-inflammatory cytokine production, the decreased tissue pathogenicity and increased survival of *Mif* ^*-/-*^ mice also could be due to a superior ability of the *Mif* ^*-/-*^ mice to mount a parasite-specific antibody response [13]. As shown in Figs 3A and S4, similar serum levels of parasite-specific IgG antibodies were recorded in WT and *Mif* ^*-/-*^ mice until 1.5 months p.i.; thereafter and from 3 months p.i., the IgG levels declined in WT mice while they remained elevated in *Mif* ^*-/-*^ mice. The drop in IgG levels in infected WT mice did not correlate with a decrease in the number of total (B220^+^) or germinal center (GL-7^+^Fas^+^B220^+^) splenic B-cells (Figs 3B and 3C, S5A and S5B), but could be due to an increase in B-cell apoptosis (Fig 3D). The increased IgG levels observed in infected *Mif* ^-/-^ mice as compared to WT mice was associated with an increased number of total and germinal center B-cells as well as with lower B-cell apoptosis (Fig 4A–4D).

Collectively, in *T*. *congolense*-infected mice, the absence of MIF results in a reduced pro-inflammatory immune response, which in turn could contribute to a lower hepato-splenomegaly and an enhanced B-cell response that collectively could enhance the survival time.

### 2. MIF deficiency correlates with reduced anemia during *T*. *congolense* infection

Anemia is the prominent pathogenic feature of a natural *T*. *congolense* infection, which is mediated by hematopoietic cells, but not by T lymphocytes or antibodies [10,29]. Since MIF (i) contributes to the accumulation of WBC (including phagocytes; Fig 1F) in tissues that are potentially involved in erythrophagocytosis and extramedullary erythropoiesis, and (ii) stimulates the production of the erythroid lineage development-blocking cytokine IL-6 [30] in the two main erythropoietic tissues (bone marrow, spleen; Fig 2B and 2C), we investigated MIF’s role in anemia development during *T*. *congolense* infection.

Anemia is characterized by two distinct phases: (i) a rapid decline in RBC levels followed by partial recovery during the early phase of infection and (ii) a more progressive decline in RBC levels during the chronic phase of infection (Fig 4A). During the early phase of infection (i.e. day 5–10 p.i.), the RBC percentages initially dropped to about 50% of non-infected mice in both WT and *Mif* ^-/-^ mice (Fig 4A). Between day 10–14 p.i., a partial recovery that reaches about 75% of the RBC level in non-infected mice occurred in WT mice, while in *Mif* ^-/-^ mice this recovery reached about 95% (Fig 4A). Subsequently, during the chronic phase of infection, the RBC levels declined progressively and remained significantly lower in WT than *Mif* ^-/-^ mice. *Mif* ^-/-^ mice also exhibited reduced anemia in a natural tsetse fly-mediated infection (S2C Fig). The serum hemoglobin and iron levels were reduced in WT mice as compared to non-infected animals during the course of infection (Fig 4B and 4C). In infected *Mif* ^-/-^ mice, these reductions were less pronounced when compared to infected WT mice. In both groups of mice, the serum hemoglobin and iron levels reached nadir levels at 3 months p.i., the time point when the differences in tissue pathogenicity were established (Figs 1C and 1D and 4A).

Collectively, during the chronic stage of *T*. *congolense* infection, MIF partially impaired recovery from early stage anemia and contributed to the decline in serum hemoglobin and iron levels.

### 3. Mif^-/-^ mice recover from dyserythropoiesis during *T*. *congolense* infection

Typically, in response to chronic anemia, splenomegaly and increased blood reticulocyte content are indicative of inefficient erythropoiesis in the bone marrow and extramedullary erythropoiesis in the spleen [31]. Compared to non-infected mice, the numbers of splenic Ter119^+^ RBCs increased more prominently in *T*. *congolense*-infected WT than *Mif* ^-/-^ mice from 3 months p.i. (Fig 4D). Remarkably, the largest increase in cell number in the spleens of infected mice was found in the RBC and not the WBC compartment (Fig 4D), showing the expansion of the erythroid compartment as a main cause for *T*. *congolense*-associated splenomegaly.

**Fig 5:**
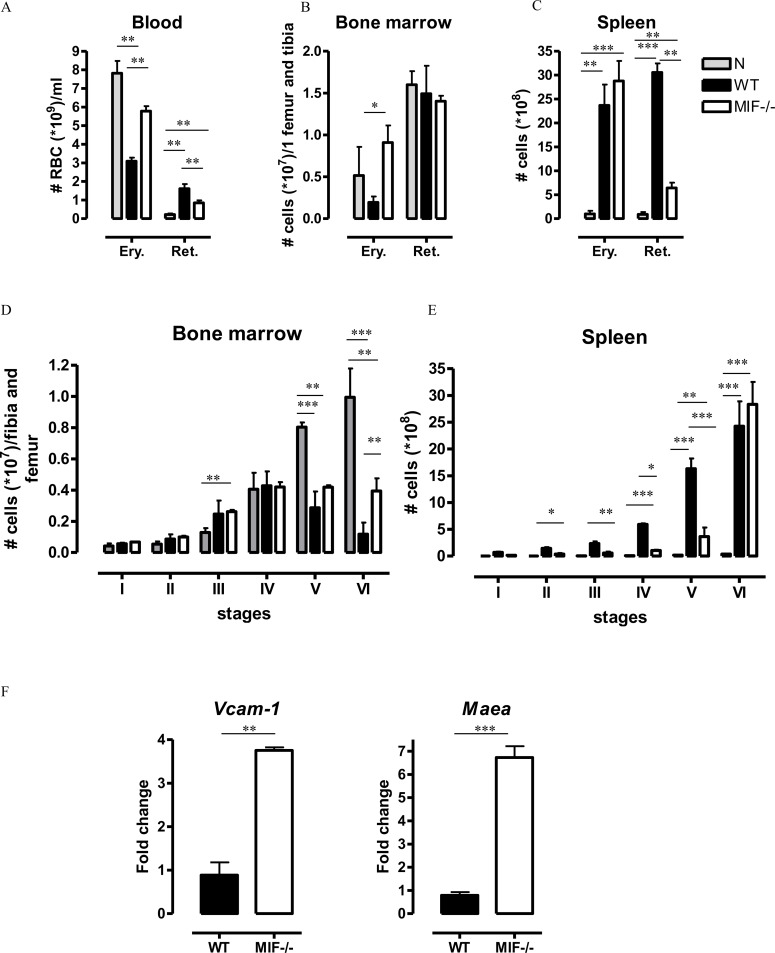
MIF contributes to dyserythropoiesis during *T*. *congolense* infection. At 3 months p.i., **(A-C)** numbers of mature RBCs (erythrocytes, Ery.) and immature RBCs (reticulocytes, Ret.) defined as described in S6A Fig in **(A)** blood, **(B)** bone marrow and **(C)** spleen of WT and *Mif* ^*-/-*^ mice. WT (black bar), *Mif* ^-/-^ (open bar) and non-infected (grey bar) mice. Results are representative of 3 independent experiments and shown as mean of 3 individual mice ± SEM. **(D, E)** Numbers of the different erythroid populations (defined as described in S6B Fig) in **(D)** bone marrow and **(E)** spleen. **(F)** Gene expression levels of *Vcam1* and *Maea* in total spleen from WT (black bar) and *Mif* ^−/−^ (white bar) mice at 3 months p.i. Gene expression levels were normalized using *S12* and expressed relatively to expression levels in non-infected mice. Results are representative of 2 independent experiments and presented as mean of 3–5 individual mice ± SEM. *: p≤0.05, **: p≤0.01, ***: p≤0.001.

The relative abundance of Ter119^+^CD71^+^ reticulocytes and mature Ter119^+^CD71^-^ RBCs (identified as described in S6A Fig) was quantified in the blood of WT and *Mif* ^-/-^ mice at 3 months p.i., which corresponds with a time point when maximal cell numbers, level of hepato-splenomegaly and tissue pathogenicity were reached in both groups of mice (Figs 1C–1F and S3). The concentration of reticulocytes increased in the blood of infected WT mice when compared to non-infected animals, and this increase was less pronounced in *Mif* ^-/-^ mice (Fig 5A). Concomitantly, the reduction in the concentration of mature RBCs was more pronounced in the blood of WT than *Mif* ^-/-^ mice. Within the erythropoietic tissues of infected WT mice, reticulocyte and mature RBC accumulation was not affected in the bone marrow, but was dramatically increased in the spleen (Fig 5B and 5C), suggesting extramedullary erythropoiesis. A comparison of infected WT and *Mif* ^*-/-*^ mice revealed a detrimental contribution of MIF to mature RBC accumulation in the bone marrow, and to reticulocyte accumulation in the spleen (Fig 5B and 5C).

Next, we assessed the stage at which erythropoiesis could be affected (from nucleated erythroblasts (I) until enucleated erythrocytes (VI)), by gating for Ter119^+^ RBCs in a CD44 versus FSC-A plot [32] (S6B Fig). In the bone marrow of infected WT mice, a blockade in the two last steps of RBC differentiation resulted in decreased percentage (S6C Fig) and numbers (Fig 5D) of nucleated reticulocytes (stage V) and enucleated reticulocyte/erythrocyte (stage VI) cells. In the spleen, infected WT mice exhibited increased accumulation of the RBC differentiation stage III polychromatic erythroblasts to stage VI mature RBCs (Fig 5E), confirming extramedullary erythropoiesis in this organ. The erythropoiesis efficacy in the bone marrow and the spleen was improved in infected *Mif* ^*-/-*^ as compared to WT mice. Indeed, *Mif* ^-/-^ mice exhibited a more efficient RBC maturation mainly during the transition from orthochromatic erythroblasts and nucleated reticulocytes (stage IV and V) to the last step of differentiation i.e. enucleation of erythrocytes (stage VI, Fig 5D and 5E). Of note, the gene expression levels of *Vcam1* (Vascular cell adhesion molecule-1) and *Maea* (EMP: erythroblast-macrophage protein), two molecules crucial for erythroblast—macrophage interaction in the terminal stage of erythropoiesis, i.e. during the erythroblast enucleation [33,34], were higher in *Mif* ^-/-^ than in WT mice (Fig 5F), suggesting a negative effect of MIF on the reticulocyte terminal maturation.

Collectively, these data indicated that mice exhibited reticulocytosis during the chronic stage of *T*. *congolense* infection, which was likely due to an increased production of RBCs, mainly in the spleen, to overcome the chronic loss of mature RBCs observed in the blood. Moreover, RBCs were impaired in their terminal stages of maturation both in the bone marrow and spleen of infected mice. Finally, MIF contributed to the reticulocytosis and to the impairment of the terminal differentiation of RBCs from the orthochromatic erythroblast to the enucleated reticulocyte/erythrocyte stage.

### 4. Mif^-/-^ mice exhibit reduced erythrophagocytosis, heme catabolism and tissue iron accumulation during *T*. *congolense* infection

**Fig 6:**
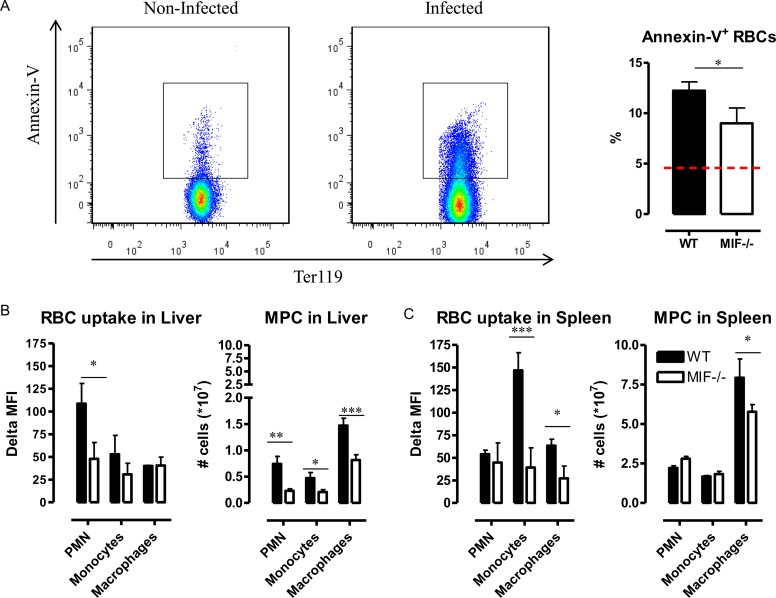
MIF contributes to erythrophagocytosis during *T*. *congolense* infection. **(A)** Representative Annexin-V gating strategy and percentage of Annexin-V^+^ RBCs in blood of WT (black bar) and *Mif* ^-/-^ (open bar) mice. **(B, C left panel)** At 3 months p.i., 10^9^ pHrodo labelled RBCs isolated from non-infected WT mice were injected i.v. in WT (black bar) or *Mif* ^-/-^ (open bar) mice. 18 h later, mice were sacrificed, liver **(B)** and spleen **(C)** myeloid phagocytic cells (MPC), namely CD11b^+^Ly6C^int^Ly6G^+^ PMNs, CD11b^+^Ly6C^high^Ly6G^-^ monocytes and CD11b^+^Ly6C^-^Ly6G^-^F4/80^+^ macrophages (identified as described in S7A Fig) were tested for delta median fluorescent intensity (MFI) of the intracellular pHrodo signal determined by subtracting the PE signal of cells from mice receiving unlabeled RBCs from the PE signal of cells from mice receiving pHrodo-labeled RBCs. **(B, C right panel)** Numbers of MPC in the **(B)** liver and **(C)** spleen of WT (black bar) or *Mif* ^-/-^ (open bar) mice. Results are representative of 2 independent experiments and presented as mean of 3 individual mice ± SEM. *: p<0.05; **: p<0.01.

The percentage of blood Annexin-V^+^ RBCs increased in *T*. *congolense*-infected WT mice, and this increase was lower in *Mif* ^*-/-*^ than in WT mice (Fig 6A). Phosphatidylserine exposure, which forms the basis of the Annexin-V staining assay, is an “eat-me” signal observed during apoptosis of senescent cells. Thus, we investigated whether an increased RBC elimination through phagocytosis in the liver and the spleen contributed to anemia in *T*. *congolense-* infected mice. An assay consisting of i.v. injection of pHrodo-labeled RBCs in infected WT or *Mif* ^-/-^ mice followed by analysis of the appearance of a fluorescent signal in phagocytes from the liver and the spleen that have engulfed labeled RBCs [35], was used. In infected WT mice, PMNs, monocytes and macrophages (gated as in S7A Fig) exhibited RBC phagocytic activity. This activity was more pronounced and MIF-dependent for PMNs in the liver and for monocytes and macrophages in the spleen (Fig 6B and 6C). In parallel, a higher number of phagocytic PMNs, monocytes and macrophages was observed in the liver of infected WT mice when compared to *Mif* ^*-/-*^ mice (Fig 6B and 6C). The same held true for macrophages in the spleen. Together, these data suggest that the reduced anemia in *T*. *congolense*-infected *Mif* ^*-/-*^ mice is a combined effect of a reduced apoptosis/senescence of RBCs, a reduced number of phagocytic cells and a reduced phagocytic capacity of these cells.

**Fig 7:**
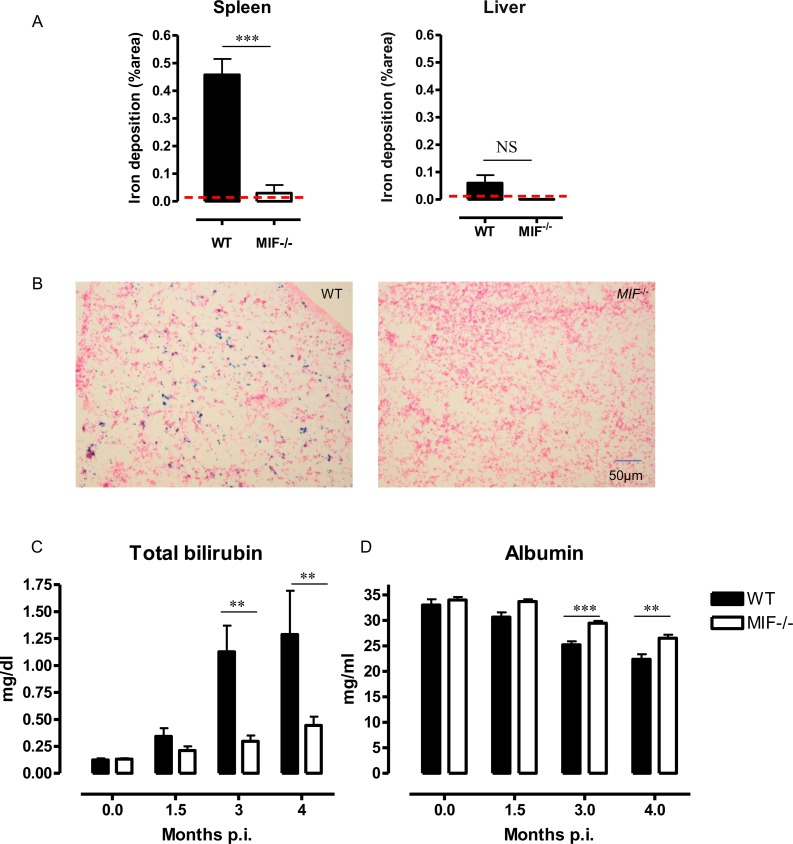
MIF contributes to heme catabolism and tissue iron accumulation during *T*. *congolense* infection. At 3 months p.i., **(A)** iron deposition was quantified on spleen and liver sections of WT (black bar) and *Mif* ^*-/-*^ (open bar) mice and expressed as % of stained area analyzed in a region of interest. Non-infected (dashed line) mice. **(B)** Representative Perl’s Prussian staining on spleen sections. **(C, D)** At 1.5, 3 and 4 months p.i., serum **(C)** total bilirubin and **(D)** albumin levels in WT and *Mif* ^*-/-*^ mice. Values represent mean ± SEM of 5 mice per group. One representative of 2 independent experiments is shown. **: p<0.01; ***: p<0.005.

Erythrophagocytosis results in the release of hemoglobin within liver and spleen phagocytes, where the heme is catabolized to iron, carbon monoxide and bilirubin. The latter is then released in the circulation and coupled to albumin to be transported to hepatocytes [36]. Accordingly, histological analyses revealed iron accumulation mainly in the splenic tissue of infected WT mice (Fig 7A and 7B). Furthermore, the observed erythrophagocytosis in *T*. *congolense*-infected WT mice coincided with a progressive increase in total bilirubin and decrease in albumin serum levels (Fig 7C and 7D). The decreased anemia and erythrophagocytosis observed in infected *Mif* ^*-/-*^ mice correlated with lower intracellular iron retention, lower bilirubinemia and higher albuminemia as compared to WT mice (Fig 7A–7D).

### 5. MIF contributes to hemodilution in *T*. *congolense*-infected mice

**Fig 8:**
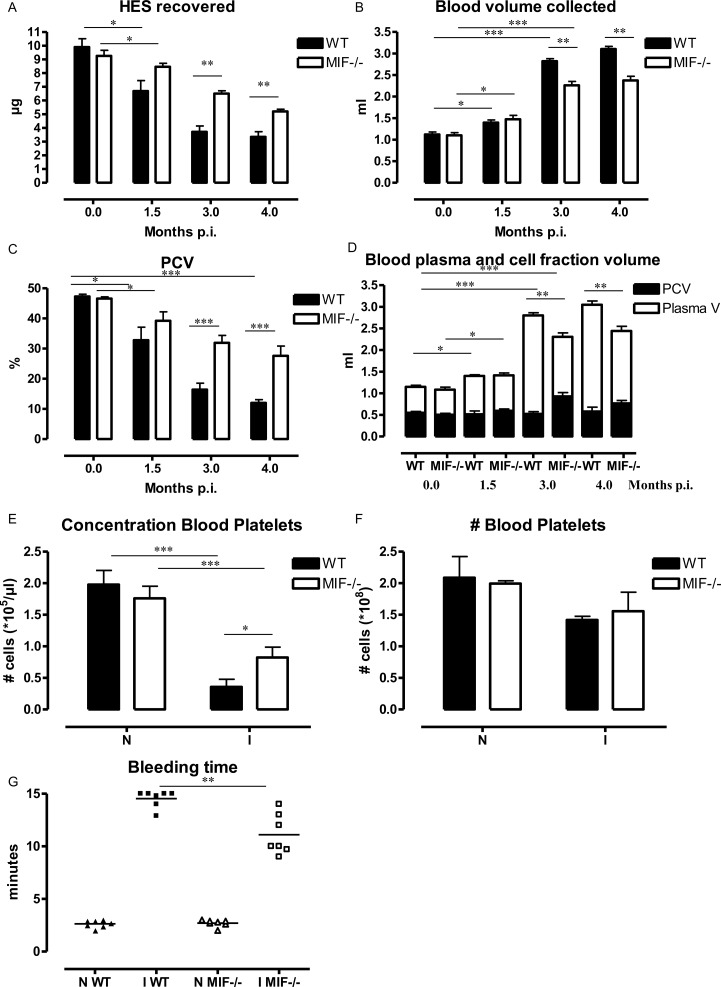
MIF contributes to hemodilution during *T*. *congolense* infection. At 1.5, 3 and 4 months p.i., 10 μg APC-conjugated hydroxyethyl starch (HES) were injected i.v. in WT (black bar) or *Mif* ^-/-^ (open bar) mice. Mice were exsanguinated 5–10 minutes later via cardiac puncture and tested for **(A)** HES concentration, **(B)** the total blood volume collected and **(C)** Pack cell volume (PCV). **(D)** Total plasma (white bar) and PCV (black bar) volumes calculated based on the total blood (B) and the % PCV (C). **(E)** Concentration of CD41^+^ platelets (gated as described in S7B Fig) was determined at 3 months p.i. **(F)** Total number of platelets in the total blood volume. **(G)** Bleeding time of non-infected (N) and 3 months infected (I) mice. For ethical reasons the bleeding of WT mice was stopped by sealing the wound after 15 minutes. Results are representative of 2 independent experiments and presented as mean of 7 individual mice ± SEM. **: p<0.01; ***: p<0.005.

Hemodilution can also contribute to anemia [37]. Moreover, the hypoalbuminemia observed in *T*. *congolense-*infected mice may also reflect hemodilution. APC-labelled hydroxyethyl starch (HES) is used to monitor hemodilution and is not affected by differences in RBC numbers. Hence, HES was injected i.v. in WT and *Mif* ^*-/-*^ mice at different time points post *T*. *congolense* infection. After 5–10 minutes, the blood was collected and the blood volume and concentration of HES were evaluated. As compared to non-infected mice, the blood HES concentration dropped in infected WT mice while the volume of the blood collected increased, whereby there was about a 3-fold change from 3 months p.i. (Fig 8A and 8B). These data suggest that hemodilution was occurring in *T*. *congolense*-infected mice during the later stage of infection. In agreement with observations in *T*. *congolense-*infected cattle [38], the packed cell volume (PCV) of the collected blood was reduced in WT mice as compared to non-infected animals (Fig 8C). However, when taking into account the blood volume in the whole animal, it appeared that the total PCV, i.e. the total amount of RBCs, was not affected by the infection while the plasma volume was increased (Fig 8D). When compared to infected WT mice, the blood and plasma volume were lower, and the HES concentration and total PCV were higher in infected *Mif* ^*-/-*^ mice (Fig 8A–8D). Together, these data indicated that anemia resulted from MIF-dependent hemodilution and not from lower production of RBCs in *T*. *congolense*-infected mice.

Hemodilution can also give rise to a reduction in platelet concentration, which in turn contributes to inefficient blood coagulation. As shown in Fig 8E, the concentration of FSC^lo^/SSC^lo^ CD41^+^ platelets (gated as in S7B Fig) declined in *T*. *congolense-*infected WT mice and to a lesser extent also in *Mif* ^*-/-*^ mice. However, when taking into consideration the total blood volume, the number of platelets per animals was not affected by the infection (Fig 8F). Furthermore, the platelet dilution was associated with a drastic increase in clotting time in infected WT mice. Indeed, while the clotting time of non-infected mice was about 2 minutes, >70% of the infected WT mice continued to bleed 15 minutes after the tail cut (time at which the wound was sealed following ethical guideline to avoid otherwise lethal hemorrhage) (Fig 8G). The coagulation time in infected *Mif* ^*-/-*^ mice was higher than in non-infected mice but remained lower than in infected WT mice, with all mice coagulating in about 10 minutes.

Collectively, these data suggest that MIF contributed to hemodilution that coincided with decreased blood platelet concentration. Combined, these effects can result in inefficient coagulation during *T*. *congolense* infection.

### 6. MIF treatment recapitulates anemia by impairing terminal erythroid differentiation and by inducing hemodilution in *T*. *congolense*-infected Mif^-/-^ mice

**Fig 9:**
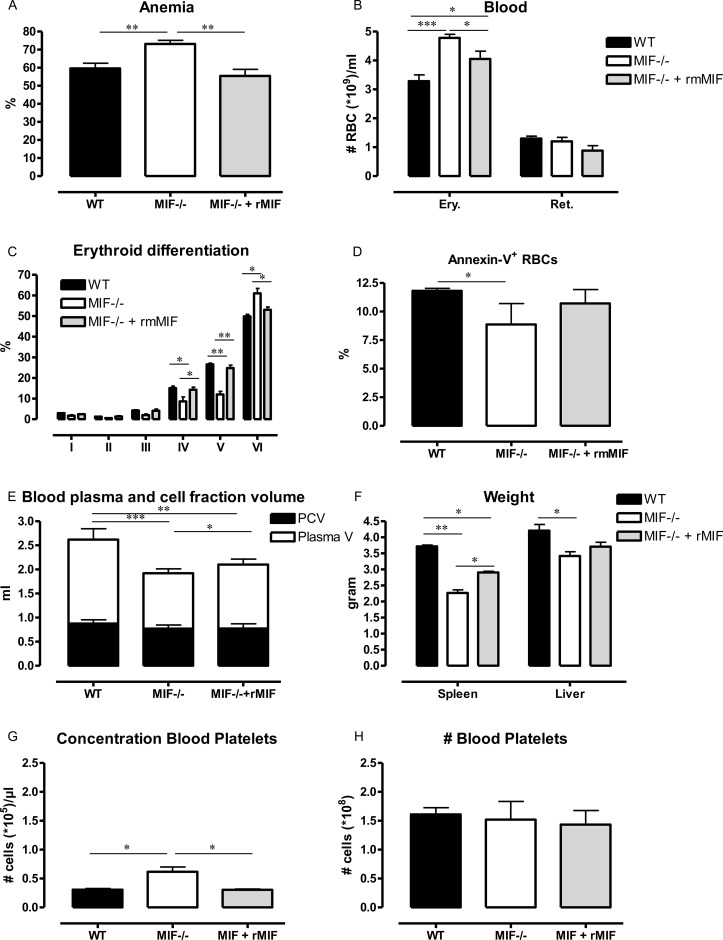
rMIF treatment in *T*. *congolense*-infected MIF^-/-^ mice recapitulates anemia and hemodilution development. At 3 months p.i., *Mif* ^-/-^ mice received 200 ng rMIF every second day for 1 week. **(A)** Percentage of RBCs in blood, whereby the number of RBCs in non-infected mice was set as 100%; **(B)** Numbers of blood mature (erythrocytes, Ery.) and immature (reticulocytes, Ret.) RBCs defined as described in S6A Fig; **(C)** Percentage of the different erythroid populations defined as described in S6B Fig in the spleen; **(D)** Percentage of blood Annexin-V^+^ RBCs gated as in Fig 6A; (E) Total plasma (white bar) and PCV (black bar) volumes calculated based on the total blood volume and the % PCV; **(F)** Spleen and liver weights; **(G, H)** Concentration and number of CD41^+^ platelets gated as described in S7B Fig in total blood volume were determined in rMIF-treated *Mif* ^-/-^ mice (grey bar) as well as in *Mif* ^-/-^ (open bar) and WT (black bar) mice. Results are representative of 2 independent experiments and presented as mean of 3–5 individual mice ± SEM. *: p≤0.05, **: p≤0.01, ***: p≤0.001.

To further assess whether MIF triggered hemodilutional anemia, *Mif* ^*-/-*^ mice at 3 months p.i. were treated with recombinant mouse MIF (rMIF) every second day for 1 week and tested for anemia-associated parameters. rMIF-treated *Mif* ^*-/-*^ mice exhibited a more severe anemia than untreated *Mif* ^*-/-*^ mice, reaching a percentage of RBC levels close to that of infected WT mice (Fig 9A). Moreover, the number of erythrocytes and reticulocytes—albeit to a non-significant extent, decreased in the blood of rMIF-treated *Mif* ^*-/-*^ mice (Fig 9B). This effect coincided with a drop in the terminal stage VI of erythroid development in the spleen, which paralleled the results of WT mice (Fig 9C), thereby strengthening the notion that MIF affected the reticulocyte enucleation process. In addition, rMIF treatment increased the level of annexin-V^+^ apoptotic RBCs (Fig 9D). Finally, infected rMIF-treated *Mif* ^*-/-*^ mice exhibited an increased plasma volume recovered from the whole animal, increased splenomegaly, and a decreased concentration but not number of platelets (Fig 9E–9H). Collectively, these data showed that rMIF treatment in *T*. *congolense*-infected *Mif* ^*-/-*^ mice could partially recapitulate the pathogenic features associated with anemia and hemodilution development in infected WT mice. These rMIF treatment data further support the conclusion that MIF exerted negative effects on anemia and hemodilution development during the later stage of *T*. *congolense* infection.

## Discussion

We have recently reported that MIF, an upstream regulator of the inflammatory response, contributed to anemia in trypanosusceptible *T*. *b*. *brucei*-infected C57Bl/6 mice [19]. As compared to *T*. *b*. *brucei*-infected mice that are locked in a type I inflammatory immune response, *T*. *congolense-*infected C57Bl/6 mice are trypanotolerant due to their ability to restrict the type I driven immune response and to mount a tissue-protective IL-10-mediated immune response in the chronic phase of infection [10,26]. Based on the observation that MIF was produced in the chronic phase of *T*. *congolense* infection, we hypothesized that this less virulent model of African trypanosomosis could allow a refined analysis of the MIF-dependent pathogenic mechanisms at play during infection-induced tissue damage and anemia.

As in trypanosusceptible mice, in the absence of MIF, the systemic and tissue-restricted production of pathogenic chemokines (CXCL1, CCL2) and cytokines (IFN-γ, TNF, IL-6, IL-12p70) was impaired during the chronic stage of *T*. *congolense* infection. MIF deficiency also limited hepato-splenomegaly and tissue destruction, including anemia. In both *T*. *b*. *brucei*- and *T*. *congolense*-infected mice, the reduced anemia in *Mif* ^*-/-*^ mice coincided with a partial recovery of serum hemoglobin and iron levels. The higher iron bioavailability partially restored erythropoiesis, which was reflected by a decreased concentration of reticulocytes and increased concentration of RBCs in the blood and the spleen of infected *Mif* ^*-/-*^ mice. The absence of MIF also improved the terminal stage of erythroid development, i.e. the differentiation from nucleated reticulocytes to enucleated RBCs. The latter effect can result from the reduced circulating IL-6 levels in infected *Mif* ^*-/-*^ mice [30]. The reduced anemia observed in *T*. *b*. *brucei*- and *T*. *congolense-*infected *Mif* ^-/-^ mice also could result in part from the better RBC recovery during the early stage of infection (up to 15 days p.i.), which in turn could require a lower erythropoietic demand during the latter stages of infection.

In cattle, anemia development can result from the extravascular destruction of RBCs due to massive erythrophagocytosis by activated macrophages in the spleen and the liver [39]. We found that enhanced erythrophagocytosis indeed occurred in both the spleen and the liver of *T*. *congolense*-infected WT mice. This phenomenon was less pronounced in *Mif* ^-/-^ mice despite their better IgG response, and likely due to the reduced number of phagocytic hepatic macrophages, Ly6C^+^ monocytes and PMNs, and of splenic macrophages. Because of the increased erythrophagocytic activity throughout *T*. *congolense* infection, a compensatory demand for increased production of RBCs was evidenced in the spleen but not in the bone marrow. This extramedullary erythropoiesis led to a massive generation and accumulation of reticulocytes and mature RBCs that could account for the splenomegaly observed in infected mice. In the absence of MIF, an increased maturation of reticulocytes to mature RBCs occurred and coincided with reduced splenomegaly and anemia. One week of rMIF treatment in infected *Mif* ^*-/-*^ mice in turn recapitulated anemia development, including splenomegaly, increased apoptosis/senescence of RBCs and impaired maturation of reticulocytes.

Our accumulated evidence argues for hemodilution, and not erythrophagocytosis, as the main contributor to the chronic anemia developing in *T*. *congolense*-infected mice in a MIF-dependent manner. Despite a study showing no increase in the blood volume in *T*. *congolense*-infected calves [40], others found a marked hypervolemia in *T*. *congolense-*infected sheep and calves [41–45]. In line with the latter observations, the blood and plasma volume was augmented in infected WT mice. However, the total number of RBCs in the blood of infected WT mice, calculated on the basis of the blood volume and the PCV, did not differ from that of non-infected mice. These data suggested that hypervolemic hemodilution developed in infected mice. They also suggested that, in infected WT mice, the increased erythropoiesis and maturation of RBCs in the spleen could compensate for both the impaired maturation of RBCs in the bone marrow and the increased clearance of RBCs through erythrophagocytosis in the liver and the spleen. Remarkably, in anemic *T*. *b*. *brucei*-infected WT mice, the blood and plasma volumes were not affected and the PCV decreased (S8 Fig), in line with observation in *T*. *b*. *brucei*-infected calves [46]. Collectively, these data support the view that a predominantly inflammatory anemia, i.e. anemia of inflammation, develops in a type I immune environment in *T*. *b*. *brucei*-infected mice [10,47], while a predominantly hemodilutional anemia occurs in a type II environment in *T*. *congolense*-infected mice. rMIF treatment in infected *Mif* ^*-/-*^ mice phenocopied the hemodilution development, confirming a MIF-dependent mechanism in infected WT mice. Hemodilution in rMIF-treated *Mif* ^*-/-*^ mice however did not reach the level observed in WT mice. Although this result could argue for the occurrence of MIF-independent mechanisms of hemodilution, we cannot exclude the possibility that rMIF treatment for a period longer than one week may be necessary.

Hemodilution could also account for the reduced concentration of platelets in the circulation of *T*. *congolense*-infected WT mice. This hemodilutional thrombocytopenia concurred with a delayed blot clotting time in infected mice that could lead to lethal haemorrhage when the tail cut for blood sampling was not sealed. Of note, thrombocytopenia was reported to occur in *T*. *congolense-*infected cattle and sheep [41,48]. Whether hemodilution of the clotting factors also accounts for the inefficient coagulation in infected animals deserves further investigation.

Despite similarly decreasing tissue pathogenicity, a difference in the pathogenic role of MIF between trypanosusceptible and trypanotolerant mice nevertheless was observed. In *T*. *b*. *brucei*-infected mice, MIF deficiency resulted in increased production of IL-10, the main anti-pathogenic cytokine in experimental African trypanosomosis, but did not affect the survival time of the infected hosts [19]. In *T*. *congolense*-infected mice, the absence of MIF had no effect on IL-10 production but resulted in prolonged survival time. Although MIF-independent mechanisms could determine the survival of *T*. *b*. *brucei*-infected mice, we could not exclude that these mice are not sufficiently responsive to IL-10 [49,50]. Alternatively, the virulence of *T*. *b*. *brucei* due to its tissue-invading capacity could be higher than that of *T*. *congolense*, which remains strictly in the blood vessels [51]. It is postulated that *T*. *b*. *brucei*- and *T*. *congolense*-infected mice die from inflammation-mediated multiple organ failure [12], but the cause of death remains unclear and may differ between the two parasite species. In this respect, a refined mechanism for the death of *T*. *congolense*-infected WT mice could be envisaged based on the data reported herein. Our results support the interpretation that in these animals, the continual and month-lasting low-grade inflammatory response drives erythrophagocytosis and that the ensuing catabolism of hemoglobin resulted in iron accumulation mainly in the spleen, followed by the enhanced release of bilirubin in the blood circulation. Importantly, hyperbilirubinemia could favour the externalisation of phosphatidylserine on RBCs observed herein and thus further contribute to erythrophagocytosis or eryptosis during *T*. *congolense* infection [36,52]. The hyperbilirubinemia and the hypoalbuminemia—with the latter resulting most likely from the hemodilution and liver damage in infected mice, could contribute to a greater degree of systemic tissue destruction, including not only the hepatic tissue but also the heart and the brain [53,54]. The severe hemodilutional anemia could also reduce cerebral oxygen delivery and further promote cerebral damage [55,56], although *T*. *congolense* is not a blood brain barrier penetrating parasite. Combined, the hyperbilirubinemia, hypoalbuminemia, and hemodilution could thus contribute importantly to the increased mortality of *T*. *congolense*-infected WT mice. Thrombocytopenia and delayed coagulation, as correlates of hemodilution, could also negatively impact the survival of infected WT mice. MIF deficiency partially alleviated iron accumulation in tissues, hyperbilirubinemia, hypoalbuminemia and defective coagulation, most likely because *T*. *congolense-*infected *Mif* ^*-/-*^ mice exhibited reduced erythrophagocytosis combined with reduced hemodilution and liver injury. Each of these effects may be contributory to the enhanced survival of *Mif* ^*-/-*^ mice. Of course, our data do not exclude the reduced inflammatory cytokine response and the better parasite-specific antibody response, both vital for the control of African trypanosomosis [13,28,57], as reasons for the enhanced survival of *T*. *congolense-*infected *Mif* ^*-/-*^ mice.

**Fig 10:**
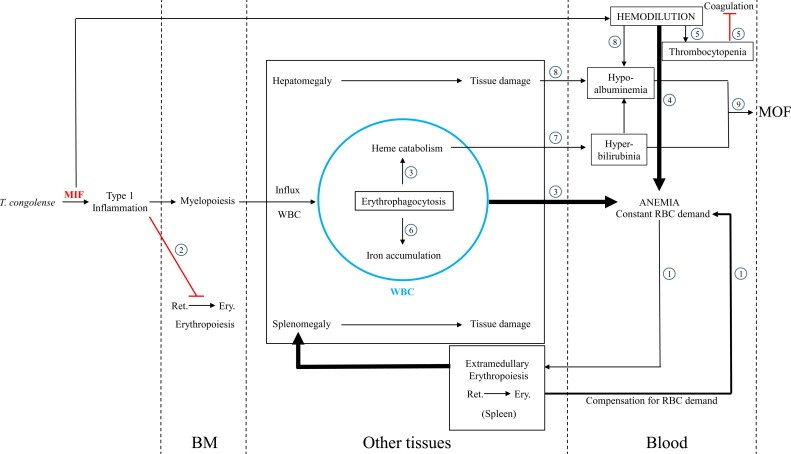
Proposed model for the contribution of MIF to pathogenic anemia during *T*. *congolense* infection in trypanotolerant C57Bl/6 mice. In the chronic phase of *T*. *congolense* infection, anemia did not result from the impaired production of mature RBCs, the total amount of RBCs being similar in infected and non-infected mice. The infection- and MIF- induced spleen extramedullary erythropoiesis (1) could thus compensate for the impaired differentiation of erythroblasts in the bone marrow (2) and for the enhanced clearance of RBCs by phagocytic cells colonizing the liver and the spleen (3). Anemia induced by *T*. *congolense* mainly occurred through hemodilution (4), that was partially mediated by MIF. Hemodilution could also account for thrombocytopenia and for impaired coagulation in infected mice (5). The heme catabolism following the chronically-induced erythrophagocytosis (3) led to iron accumulation in myeloid cells (6) and to hyperbilirubinemia (7). The combination of liver injury and hemodilution resulted in declined serum albumin levels (8) in infected mice, thereby preventing efficient removal of toxic molecules from the circulation, including bilirubin. This could cause multiple organ failure (MOF, 9) that culminates in reduced survival of the infected host.

Collectively, our results suggest that during the chronic phase of *T*. *congolense* infection, anemia did not result from the impaired production of mature RBCs. MIF-induced splenic extramedullary erythropoiesis could compensate for the impaired differentiation of erythroblasts in the bone marrow and for the enhanced erythrophagocytosis in the liver and the spleen (Fig 10). In contrast, anemia induced by *T*. *congolense* mainly occurred through MIF-dependent hemodilution. The heme catabolism ensuing erythrophagocytosis could lead to iron accumulation in tissue and to hyperbilirubinemia. Hypoalbuminemia resulting from hemodilution in infected mice impaired the elimination of toxic circulating molecules, including bilirubin. Hemodilution with thrombocytopenia as a consequence could also account for impaired coagulation in infected mice. Combined, these effects could trigger multiple organ failure and uncontrolled bleeding hereby reducing the survival time of infected mice. Together, this study suggests that interfering with MIF signaling could represent an approach to limit inflammation-associated anemia complications during natural *T*. *congolense* trypanosomosis. Given that polymorphisms in the human *MIF* gene contribute to differences in susceptibility in several inflammatory diseases [58,59], it could be interesting to assess whether differences between trypanosusceptible and trypanotolerant cattle associates with genetically-predetermined differences in MIF expression.

## Materials and Methods

### Ethics statement

All experiments, maintenance and care of the mice complied with the European Convention for the Protection of Vertebrate Animals used for Experimental and Other Scientific Purposes guidelines (CETS n° 123) and were approved by the Ethical Committee for Animal Experiments (ECAE) at the Vrije Universiteit Brussel (Permit Numbers: 14-220-4 and 14-220-6). Infection of tsetse flies with *T*. *congolense* parasites was performed in compliance with the regulations for bio-safety and under approval from the Environmental administration of the Flemish government (Permit number: BM2012-6).

### Parasites, mice and infections

Clonal *T*. *congolense* parasites (Tc13) were kindly provided by Dr. Henry Tabel (University of Saskatchewan, Saskatoon) and stored at -80°C. Wild type (WT) C57Bl/6 mice were obtained from Janvier. MIF deficient (*Mif* ^-/-^) C57Bl/6 mice generated as described in [60] were bred in our animal facility. Female mice (7–8 weeks old) were infected intraperitoneally (i.p.) with 2 x 10^3^ Tc13 trypanosomes. Alternatively, tsetse fly infection was achieved by feeding teneral flies on *T*. *congolense*-infected mice as described in [19]. When required, infected mice were treated i.p. with 200 ng of mouse recombinant MIF (rMIF, Abcam, ab191658) every second day for 1 week. Parasite and red blood cell (RBC) numbers in blood were determined via hemocytometer by cold tail-cut (2.5 μl blood in 500 μl RPMI). Anemia was expressed as the percentage of reduction in RBC counts compared to non-infected animals. Packed cell volume (PCV) was measured following collection of anti-coagulated blood in heparinized capillaries and centrifugation at 9500g for 7 min. using a micro-centrifuge (Fisher BioBlock Scientific).

### Cell isolation protocol

Liver cell isolation was performed as described by Stijlemans et al. [61]. Briefly, livers from CO_2_ euthanized mice were perfused with 30 ml heparinized saline (10 units/ml; Leo Pharma) containing 0.05% collagenase type II (*Clostridium histolyticum*; Sigma-Aldrich), excised and rinsed in saline. Following mincing in 10 ml digestive media (0.05% collagenase type II in Hanks' Balanced Salt Solution (HBSS) without calcium or magnesium; Invitrogen) and incubation at 37°C for 30 min., the digested liver was homogenized and filtered (40 μm pore filter). The cell suspension was centrifuged (7 min., 300×*g*, 4°C) and the pellet treated with erythrocyte-lysis buffer. Following centrifugation (7 min., 300×*g*, 4°C) the pellet was resuspended in 2–5 ml RPMI/5% FCS medium, cells counted and adjusted at 10^7^ cells/ml for flow-cytometric analysis and cell culturing.

Spleen and bone marrow (tibia and femur) cells were obtained by homogenizing the organs in 10 ml RPMI/5%FCS medium, passing the suspension through a 40 μm pore filter and centrifugation (7 min., 300×*g*, 4°C). Cells were counted and brought at 10^7^ cells/ml in RPMI/5% FCS medium for RBC analysis via flow cytometry. Remaining cells were pelleted (7 min., 300×*g*, 4°C) and subsequently treated with erythrocyte-lysis buffer and processed as described for the liver (see above) for analysis of white blood cells (WBCs).

### Cell culturing

Cells were diluted at 2x10^6^ cells/ml in complete medium (RPMI-1640 medium, 10% FBS, 1% sodium pyruvate (Gibco), 1% non-essential amino acids (Gibco), 1% glutamate, 1% penicillin-streptomycin). Next, 500 μl of cell suspension/well were cultured (36–48 hours, 37°C, 5% CO_2_) in 48 well plates (Nunc) and the supernatant was tested in ELISA.

### Flow cytometry

To analyze the RBC composition and platelet counts, the blood, spleen and bone marrow cells were analysed omitting RBC lysis. Briefly, total blood (2.5 μl diluted in 500 μl RPMI/5% FCS) and 10^6^ spleen or bone marrow cells (in 100 μl) were incubated (15 min., 4°C) with Fc-gamma blocking antibody (2.4G2, BD Biosciences), and subsequently stained with labelled antibodies (S1 Table) and matching control antibodies. Samples were washed with PBS, measured on FACSCanto II (BD Bioscience) and results were analysed using FlowJo software by excluding CD45^+^ cells and gating on Ter-119^+^ or CD41^+^ cells.

The WBC composition within the bone marrow, spleen and liver cells and the B cell compartment in the spleen (10^6^ cells/100 μl) were analyzed after RBC lysis as described above using labelled antibodies (S1 Table). The results were analysed after selection of CD45^+^ cells, followed by exclusion of aggregated and death cells (7AAD^+^, BD Pharmingen). Annexin-V staining was performed as described by the suppliers (TACS Annexin-V FITC Apoptosis Detection kit, R&D Systems).

### Cytokine, chemokine and antibody analysis

Blood was collected from CO_2_ euthanized mice via cardiac puncture, centrifuged (15 minutes, 10.000xg, 25°C), and serum was kept at -20°C. Serum levels of IFN-γ, IL-6, IL-10, IL-12p70, TNF and KC (CXCL1) were determined using the V-PLEX Custom Mouse Cytokine assay (Meso Scale Discovery, Maryland, USA). Serum MIF levels were measured by ELISA as recommended by the suppliers (R&D Systems). Alternatively, culture medium concentrations of MIF, TNF, CCL2 and KC (R&D Systems) as well as IFN-γ, IL-6 and IL-10 (Pharmingen) were determined by ELISA as recommended by the suppliers. Parasite-specific IgG responses were determined using soluble lysate freshly prepared from DEAE-purified *T*. *congolense* parasites recovered from WT mice at the peak of infection (around day 7). Lysate was coated overnight at 10 μg/ml PBS in 96-well Maxisorp plates (NUNC). Plates were washed (0.1% Tween 20 in PBS) and blocked (1% BSA in PBS) for 1 hour. Next, plates were washed and the sera (100 μl) serially diluted starting from 1/100 in blocking buffer were added. The ELISA was subsequently performed as described by the suppliers (SBA Clonotyping system-HRP kit (SouthernBiotech, USA)). As negative controls, blood samples incubated on lysate-free plates were used. The OD_450nm_ recorded on lysate-free plates was subtracted from the OD_450nm_ recorded on lysate-coated plates.

### Real-time quantitative polymerase chain reaction (RT-QPCR) analysis

One μg of total RNA prepared from 10^7^ cells (RNeasy plus mini kit, Qiagen) was reverse-transcribed using oligo(dT) and Superscript II Reverse Transcription following the manufacturer's recommendations (Roche Molecular Systems). RT-QPCR was performed in an iCycler iQ, with iQ SYBR Green Supermix (Bio-Rad). Primer sequences were *Vcam-1*-F: 5’-CTCTCCCAGGAATACAACGA-3’, *Vcam-1*-R: 5’-CACGTCAGAACAACCGAATC-3’ and *Maea*-F: 5’-GAGTGGTCTCCTCTCAACAG-3’, *Maea*-R: 5’-AGCTACCATCTGTC TGGATG-3’. PCR cycles consisted of 1-minute denaturation at 94°C, 45-second annealing at 55°C, and 1-minute extension at 72°C. Fold change in gene expression was expressed as compared to non-infected animals after normalization against the Ct value of the ribosomal S12 (*Mrps12*) protein as household gene.

### Erythrophagocytosis assay

The pHrodo-labeling of red blood cells (RBCs) was described in [35]. 10^9^ pHrodo-labelled RBCs isolated from non-infected WT mice were injected i.v. in WT or *Mif* ^-/-^ mice. 18 h later, mice were sacrificed, liver and spleen CD11b^+^Ly6C^int^Ly6G^+^ PMNs, CD11b^+^Ly6C^high^Ly6G^-^ monocytes and CD11b^+^Ly6C^-^Ly6G^-^F4/80^+^ macrophages were tested for delta median fluorescent intensity (MFI) of the intracellular pHrodo signal determined by subtracting the PE signal of cells from mice receiving unlabeled RBCs from the PE signal of cells from mice receiving pHrodo-labeled RBCs.

### Aspartate transaminase (AST) and alanine transaminase (ALT) measurement

Serum AST and ALT levels were determined as described by the suppliers (Boehringer Mannheim Diagnostics).

### Hemodilution assay

100 μl of APC-labelled hydroxyethyl starch (APC-HES (130/0.4) at 100 μg/ml in 0.9% NaCl) was injected i.v. (as described in [62]). After 5–10 min., blood was collected and the APC signal measured via cytofluorimeter (OD_660nm,_ Ultra Microplate reader, ELx808, Bio-Tek instruments.inc). A standard curve consisting of a serial dilution of APC-HES (starting from 200 μg/ml) diluted in blood from non-infected mice was used to calculate the concentration of APC-HES in collected blood. The OD_660nm_ from blood of non-infected mice was subtracted from all samples.

### Measurement of serum hemoglobin, iron, bilirubin and albumin content

For hemoglobin quantification, 2 μl of blood collected via cold tail cut was diluted in 200 μl distilled water in a 96 well round bottom plate (Falcon). After incubation for 30 min. at 37°C and centrifugation (600xg, 10 min.), the supernatant was collected and the OD_550nm_ measured. The hemoglobin concentration was calculated using a standard (Sigma) curve. Total iron (IRON FZ kit, Chema Diagnostics), bilirubin and albumin (Chema Diagnostica, Italy and Sigma Aldrich, respectively) were measured as recommended by the suppliers.

### Bleeding time

Bleeding times of mice were obtained by using the tail-cut model [63]. Briefly, anesthetized animals were transected at the 5-mm mark from the tip of the tail and incubated in warm saline (37°C). The time for cessation of bleeding was recorded. The experiment was terminated after 15 min. to avoid lethality, whereby the tail was cauterized and the bleeding time was taken as 15 min.

### Pearls Staining (Prussian Blue)

Total spleens and the largest lobe of the liver were embedded in Tissue-Tek O.C.T. compound (Sakura Belgium B.V.B.A.) and kept at -80°C. Next, 5 μm cryosections were cut using a Leica microtome, fixed in cold acetone for 15 min. and washed shortly in distilled water (2–3 changes). The sections were stained for 20 min. in equal volumes of warm HCl 4% and K_4_[Fe(CN)_6_]·3H_2_O 4% at 45°C. After washing shortly 3x with distilled water, the slides were counterstained with NFR (Nuclear Fast Red, Sigma-Aldrich) for 5 min. After rinsing 3x with distilled water, the slides were dehydrated (short in EtOH 96%, short in EtOH 100% (2x), 3 min. in xylol (2x)) and mounted with DPX mounting medium (Sigma-Aldrich). Images were obtained using an OLYMPUS BX41 fluorescent microscope. The CellSens Dimension 1.9 software was used for quantification. For each sample, an average quantification of 5 representative images was determined. The results are expressed as % stained area within the region of interest.

### Statistical analysis

The GraphPad Prism software was used for statistical analyses (Two-way ANOVA or student *t*-test). Values are expressed as mean ± SEM. Values of p≤ 0.05 are considered significant.

## Supporting Information

S1 Table Fluorescently labeled antibodies used. (DOCX)

S1 Fig MIF serum levels during the course of *T*. *congolense* infection. MIF serum levels during the course of *T*. *congolense* infection in C57Bl/6 mice. Results are representative of 3 independent experiments and presented as mean of 6 individual mice ± SEM, *: p≤0.05, **: p≤0.01, ***: p≤0.001.
(TIF)

S2 Fig MIF deficiency confers partial protection against a tsetse fly-based *T*. congolense infection. **(A)** Parasitemia, **(B)** survival time and **(C)** anemia during the course of infection in C57Bl/6 mice. Wild type (WT, black symbol); *Mif* ^-/-^ (white symbol) mice. Results are representative of 2 independent experiments and presented as mean **(A, C)** or median **(B)** of 6 individual mice ± SEM, **: p≤0.01.
(TIF)

S3 Fig MIF deficiency reduces hepatosplenomegaly during *T*. *congolense* infection. Representative picture of livers and spleens from *T*. *congolense-*infected (3 months p.i.) WT and *Mif* ^-/-^ mice. There was no difference in liver and spleen size between non-infected WT and *Mif* ^-/-^ mice.
(TIF)

S4 Fig MIF negatively affects anti-trypanosome IgG responses during *T*. *congolense* infection. At 1.5, 3 and 4 months p.i., the serum IgG antibody titers from WT (black box) and *Mif* ^-/-^ (white box) mice were determined by ELISA on lysates from *T*. *congolense* parasites. The serum was ½ serially diluted starting from a 1/100 dilution and the OD_450nm_ (subtracting background OD_450nm_ of lysate-free ELISA) was plotted. In parallel, the anti-trypanosome IgG level of non-infected mice also was determined. Results are representative of 2 independent experiments and presented as median of 3–4 individual mice ± SEM.
(TIF)

S5 Fig Representative gating strategy used to identify B-cells, apoptotic B-cells and germinal center B-cells. At 3 months p.i., **(A)** representative gating strategy used to identify splenic mature B-cells: CD45^+^ cells and singlet cells were gated followed by selection of B220^+^MHC-II^+^ B-cells. The percentage of Annexin-V^+^ B-cells is shown in histogram for non-infected (black line) and infected (blue line) WT and *Mif* ^-/-^ mice. **(B)** Within the splenic B220^+^MHC-II^+^ cells, the germinal center GL-7^+^Fas^+^ B-cells were identified in non-infected (left panel), WT (middle panel) and *Mif* ^-/-^ (right panel) mice.
(TIF)

S6 Fig Representative gating strategy used to discriminate mature and immature RBCs, and the different stages of erythroid development. At 3 months p.i., **(A)** representative gating strategy used to identify mature RBCs (erythrocytes, Ery.) and immature RBCs (reticulocytes, Ret.) in bone marrow. Within CD45^-^ cells gated from a FSC-A/SSC-A plot, Ter-119^+^ cells were selected and displayed in a histogram. CD71 expression was then used to discriminate mature RBCs (erythrocytes (Ery.), Ter-119^+^ CD71^-^) and immature (reticulocytes (Ret.), Ter-119^+^ CD71^+^) RBCs. **(B**) 7AAD^-^CD45^-^Ter-119^+^ cells were plotted in a CD44/FSC-A plot to identify nucleated erythroblasts (pro (I), basophilic (II), polychromatic (III, orthochromatic (IV) erythroblasts), from nucleated reticulocytes (V) and enucleated erythrocytes (VI). **(C)** Representative percentage of the different erythroid populations in the bone marrow (upper panels) and spleen (lower panels) of non-infected (naive, left panel), infected WT (middle panel) and infected *Mif* ^-/-^ (right panel) mice.
(TIF)

S7 Fig Representative gating strategy used to discriminate PMNs, Ly6C^high^ monocytes, macrophages and platelets. At 3 months p.i., **(A)** following gating on 7AAD^-^ cells, CD45^+^ cells were displayed in a CD11b/CD31 plot to out-gate endothelial cells. A Ly6C/Ly6G plot allowed to distinguish within the CD11b^+^ population Ly6C^high^Ly6G^-^ inflammatory monocytes and CD11b^+^Ly6C^int^Ly6G^+^ neutrophils (PMNs). The CD11b^+^Ly6C^int/-^Ly6G^-^ population was tested for F4/80 expression to define macrophages. **(B)** For platelet identification, small cells were selected in a SSC-A/FSC-A plot and then analyzed in a CD41/CD11b plot to identify CD41^+^ platelets.
(TIF)

S8 Fig Blood and plasma volumes during *T*. *b*. *brucei* infection. At 1 month p.i., WT (black bar) or *Mif* ^-/-^ (open bar) mice were exsanguinated via cardiac puncture and tested for **(A)** the total blood volume collected and **(B)** Pack cell volume (PCV). **(C)** Total plasma (white bars) and PCV (black bars) volumes are calculated based on the total blood (A) and the % PCV (B). Non-infected mice (N), infected mice (I). Results are representative of 2 independent experiments and presented as mean of 5 individual mice ± SEM, **: p≤0.01, ***: p≤0.001.
(TIF)
