# Supplementary material for: MIF-Mediated Hemodilution Promotes Pathogenic Anemia in Experimental African Trypanosomosis
Source: PLoS Pathog. 2016 Sep 15;12(9):e1005862. doi: 10.1371/journal.ppat.1005862 (PMC5025191; doi:10.1371/journal.ppat.1005862)
Supplement: S1 Table — (DOCX) [file ppat.1005862.s001.docx]

**Table S1: Fluorescently labeled antibodies used**

| Antibody | Fluorescent label | Company |
| --- | --- | --- |
| Ter-119 | PE  APC | eBioscience  eBioscience |
| CD71 | FITC | eBioscience |
| CD44 | APC | eBioscience |
| CD41 | FITC | BD Pharmingen |
| CD45 | APC-Cy7 | BD Pharmingen |
| CD11b | PE-Cy7 | BD Pharmingen |
| CD31 | APC | eBioscience |
| Ly6C | Pacific Blue | BD Pharmingen |
| Ly6G | Per-CP-Cy5.5 | BD Pharmingen |
| F4/80 | PE | BD Pharmingen |
| B220 | AmCyan | BD Pharmingen |
| MHC-II | Per-CP-Cy5.5  Pacific Blue | BD Pharmingen  BD Pharmingen |
| GL-7 | APC | BD Pharmingen |
| Fas | PE-Cy7 | eBioscience |
