# Supplementary figures and images for: MIF-Mediated Hemodilution Promotes Pathogenic Anemia in Experimental African Trypanosomosis
Source: PLoS Pathog. 2016 Sep 15;12(9):e1005862. doi: 10.1371/journal.ppat.1005862 (PMC5025191; doi:10.1371/journal.ppat.1005862)

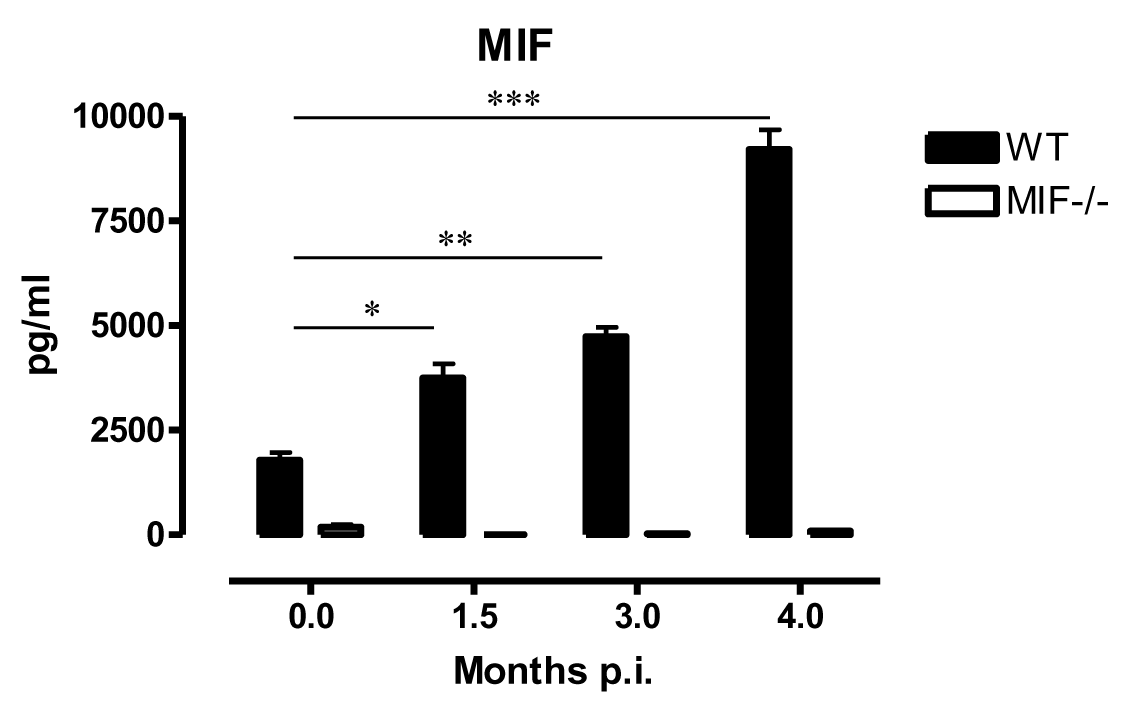

Supplement: S1 Fig — MIF serum levels during the course of T. congolense infection in C57Bl/6 mice. Results are representative of 3 independent experiments and presented as mean of 6 individual mice ± SEM, *: p≤0.05, **: p≤0.01, ***: p≤0.001. (TIF) [file ppat.1005862.s002.tif]

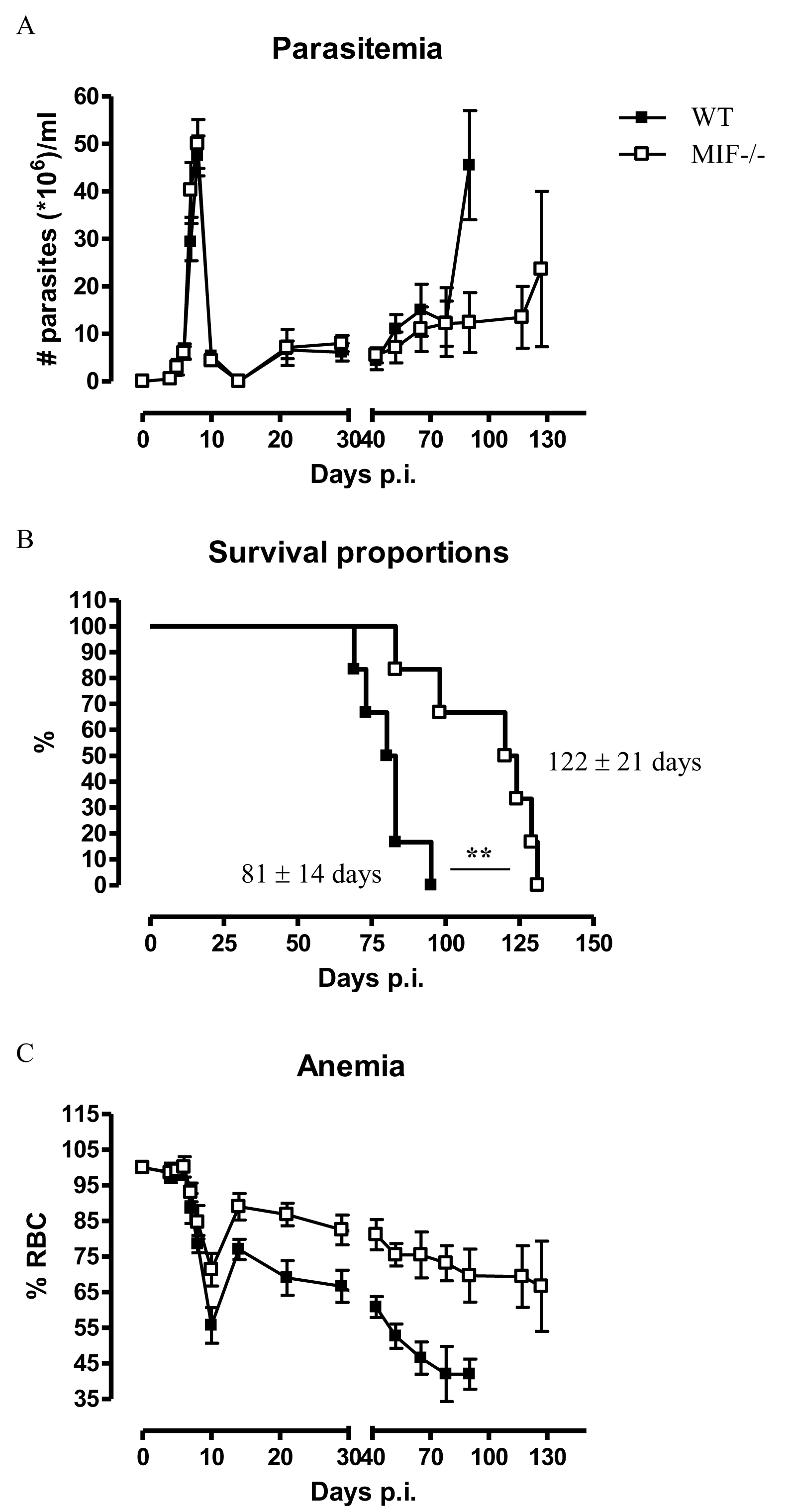

Supplement: S2 Fig — (A) Parasitemia, (B) survival time and (C) anemia during the course of infection in C57Bl/6 mice. Wild type (WT, black symbol); Mif -/- (white symbol) mice. Results are representative of 2 independent experiments and presented as mean (A, C) or median (B) of 6 individual mice ± SEM, **: p≤0.01. (TIF) [file ppat.1005862.s003.tif]

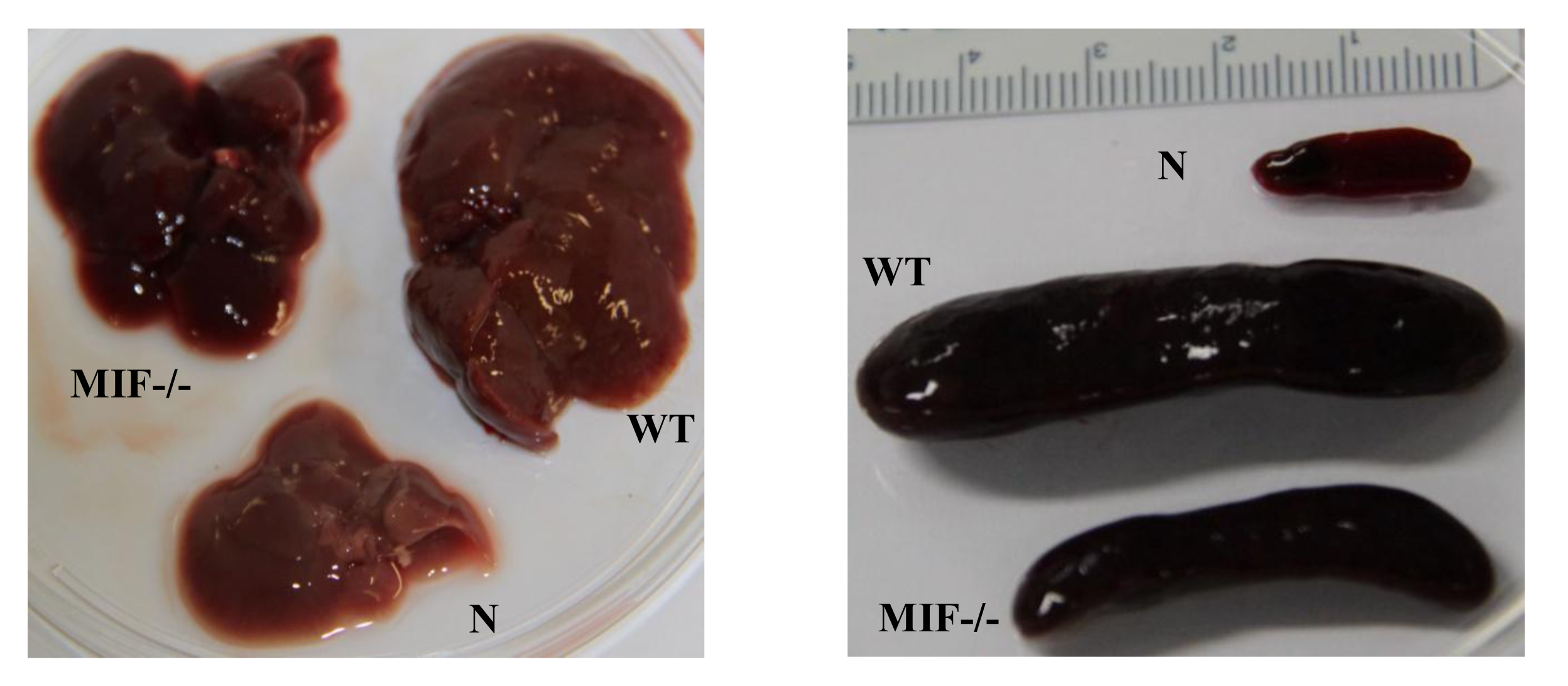

Supplement: S3 Fig — Representative picture of livers and spleens from T. congolense-infected (3 months p.i.) WT and Mif -/- mice. There was no difference in liver and spleen size between non-infected WT and Mif -/- mice. (TIF) [file ppat.1005862.s004.tif]

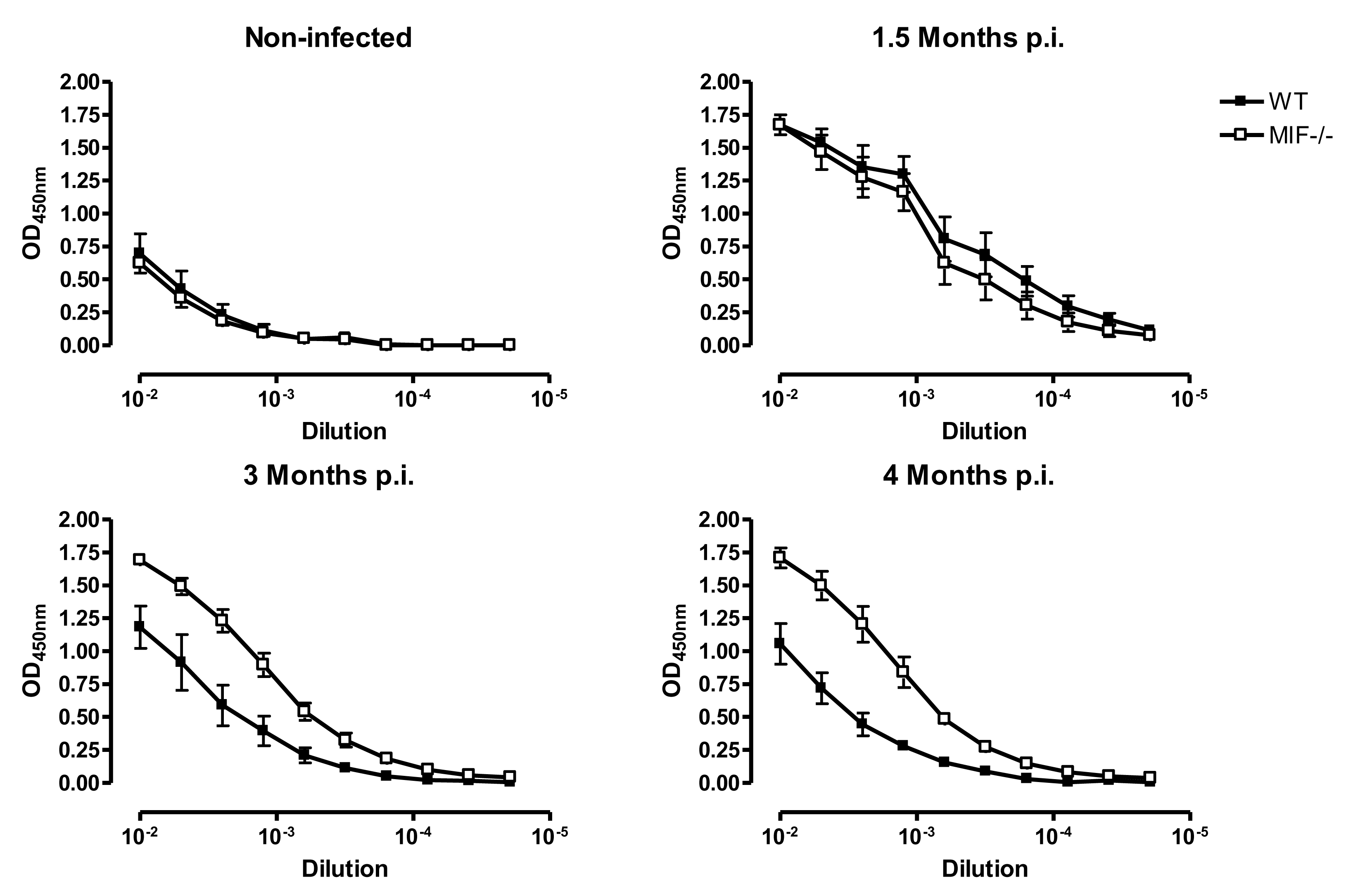

Supplement: S4 Fig — At 1.5, 3 and 4 months p.i., the serum IgG antibody titers from WT (black box) and Mif -/- (white box) mice were determined by ELISA on lysates from T. congolense parasites. The serum was ½ serially diluted starting from a 1/100 dilution and the OD450nm (subtracting background OD450nm of lysate-free ELISA) was plotted. In parallel, the anti-trypanosome IgG level of non-infected mice also was determined. Results are representative of 2 independent experiments and presented as median of 3–4 individual mice ± SEM. (TIF) [file ppat.1005862.s005.tif]

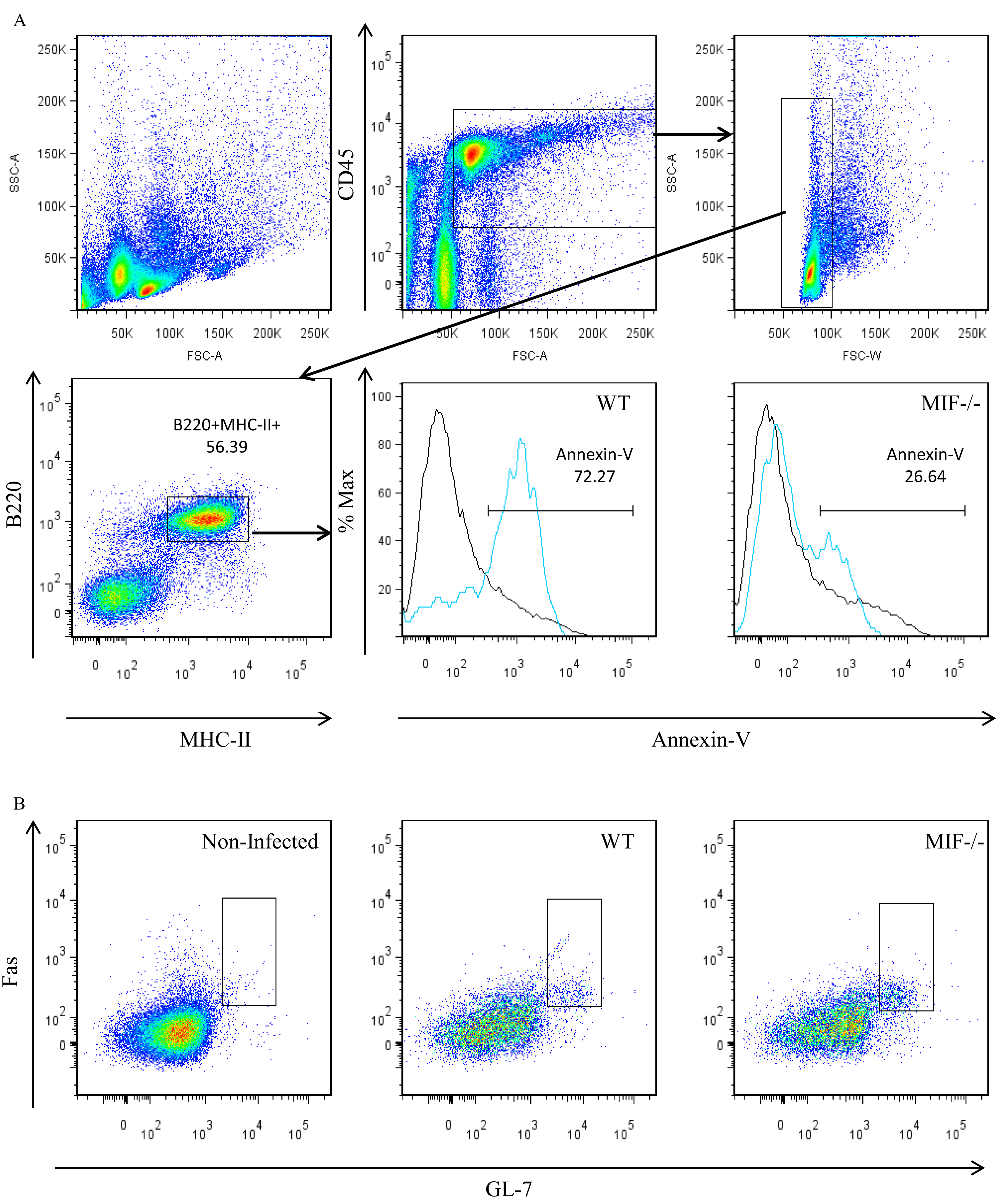

Supplement: S5 Fig — At 3 months p.i., (A) representative gating strategy used to identify splenic mature B-cells: CD45+ cells and singlet cells were gated followed by selection of B220+MHC-II+ B-cells. The percentage of Annexin-V+ B-cells is shown in histogram for non-infected (black line) and infected (blue line) WT and Mif -/- mice. (B) Within the splenic B220+MHC-II+ cells, the germinal center GL-7+Fas+ B-cells were identified in non-infected (left panel), WT (middle panel) and Mif -/- (right panel) mice. (TIF) [file ppat.1005862.s006.tif]

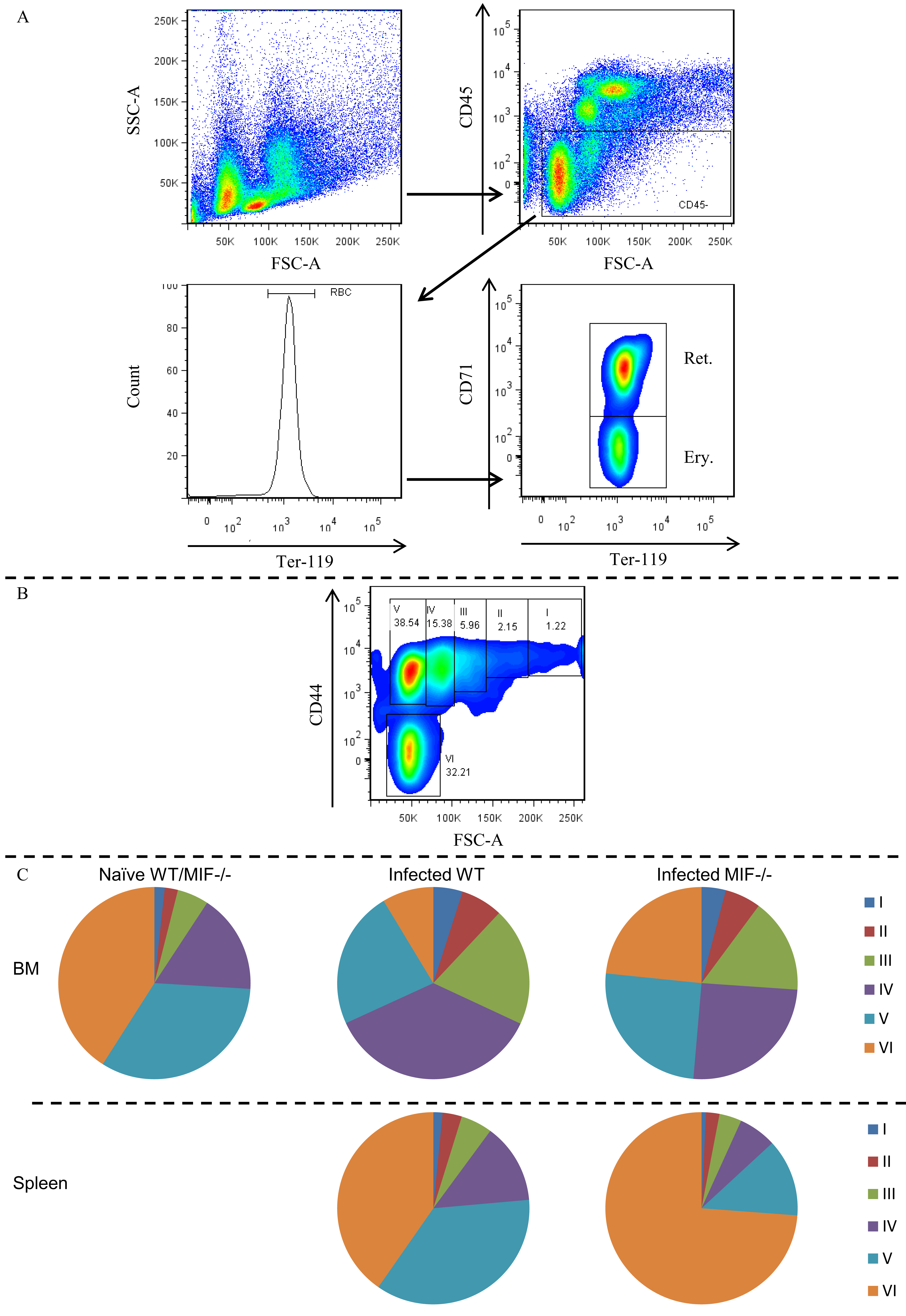

Supplement: S6 Fig — At 3 months p.i., (A) representative gating strategy used to identify mature RBCs (erythrocytes, Ery.) and immature RBCs (reticulocytes, Ret.) in bone marrow. Within CD45- cells gated from a FSC-A/SSC-A plot, Ter-119+ cells were selected and displayed in a histogram. CD71 expression was then used to discriminate mature RBCs (erythrocytes (Ery.), Ter-119+ CD71-) and immature (reticulocytes (Ret.), Ter-119+ CD71+) RBCs. (B) 7AAD-CD45-Ter-119+ cells were plotted in a CD44/FSC-A plot to identify nucleated erythroblasts (pro (I), basophilic (II), polychromatic (III, orthochromatic (IV) erythroblasts), from nucleated reticulocytes (V) and enucleated erythrocytes (VI). (C) Representative percentage of the different erythroid populations in the bone marrow (upper panels) and spleen (lower panels) of non-infected (naive, left panel), infected WT (middle panel) and infected Mif -/- (right panel) mice. (TIF) [file ppat.1005862.s007.tif]

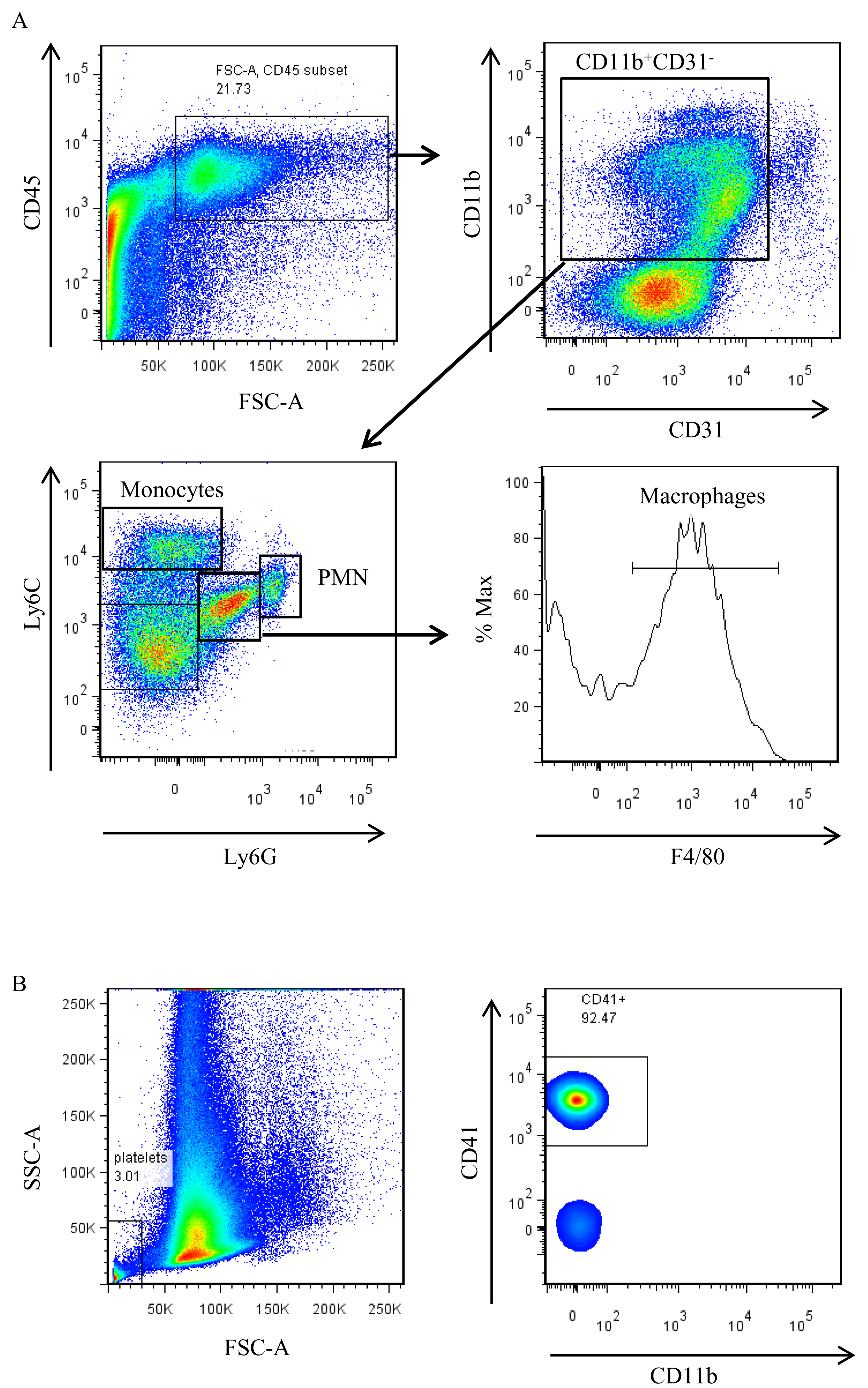

Supplement: S7 Fig — At 3 months p.i., (A) following gating on 7AAD- cells, CD45+ cells were displayed in a CD11b/CD31 plot to out-gate endothelial cells. A Ly6C/Ly6G plot allowed to distinguish within the CD11b+ population Ly6ChighLy6G- inflammatory monocytes and CD11b+Ly6CintLy6G+ neutrophils (PMNs). The CD11b+Ly6Cint/-Ly6G- population was tested for F4/80 expression to define macrophages. (B) For platelet identification, small cells were selected in a SSC-A/FSC-A plot and then analyzed in a CD41/CD11b plot to identify CD41+ platelets. (TIF) [file ppat.1005862.s008.tif]

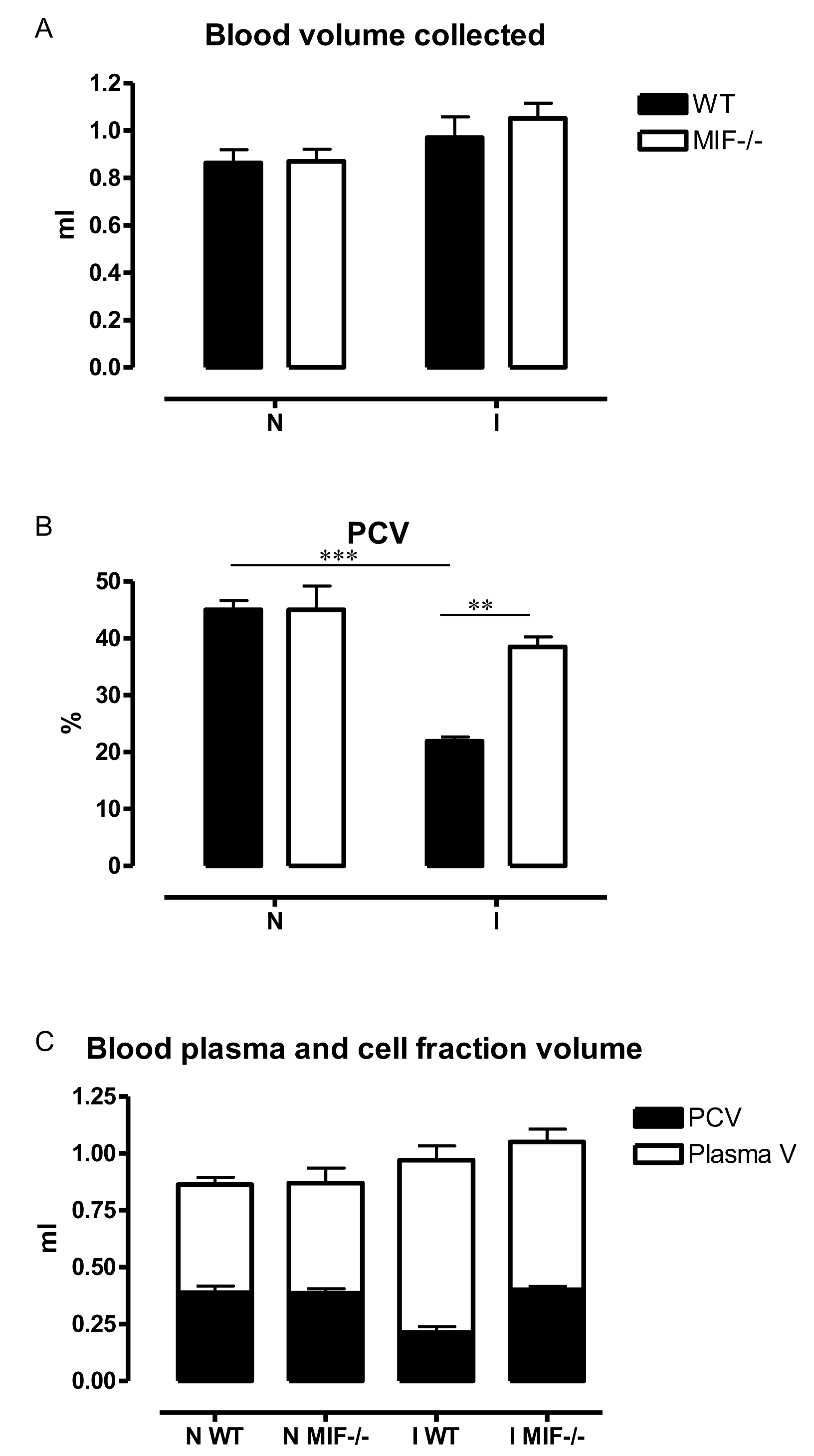

Supplement: S8 Fig — At 1 month p.i., WT (black bar) or Mif -/- (open bar) mice were exsanguinated via cardiac puncture and tested for (A) the total blood volume collected and (B) Pack cell volume (PCV). (C) Total plasma (white bars) and PCV (black bars) volumes are calculated based on the total blood (A) and the % PCV (B). Non-infected mice (N), infected mice (I). Results are representative of 2 independent experiments and presented as mean of 5 individual mice ± SEM, **: p≤0.01, ***: p≤0.001. (TIF) [file ppat.1005862.s009.tif]
